# A novel mutation located in the intermembrane space domain of *AFG3L2* causes dominant optic atrophy through decreasing the stability of the encoded protein

**DOI:** 10.1038/s41420-022-01160-9

**Published:** 2022-08-15

**Authors:** Lin Yang, Xiuxiu Jin, Ya Li, Qingge Guo, Mingzhu Yang, Ya You, Shun Yao, Xiaoli Zhang, Zhongfeng Wang, Bo Lei

**Affiliations:** 1 Zhengzhou University People’s Hospital, Henan Provincial People’s Hospital, Zhengzhou, Henan China; 2 Academy of Medical Sciences, Zhengzhou University, Zhengzhou, Henan China; 3 Henan Eye Institute, Henan Eye Hospital, Henan Provincial People’s Hospital, Zhengzhou, Henan China; 4 State Key Laboratory of Medical Neurobiology and MOE Frontiers Center for Brain Science, Institutes of Brain Science, Fudan University, Shanghai, China

**Keywords:** Disease genetics, Genome

## Abstract

Dominant optic atrophy (DOA) is the most common hereditary optic neuropathy. Although DOA is caused by mutations in several genes, there are still many cases that have not been diagnosed or misdiagnosed. Herein, we present a large family of 11 patients with DOA. To identify potential pathogenic mutations, whole exome sequencing (WES) was performed on the proband, a 35-year-old woman. WES revealed a novel pathogenic mutation (c.524T>C, p.F175S) in the *AFG3L2* intermembrane space domain, rather than in the ATPase domain, which is the hot mutation region associated with most of the previously reported DOA cases. Functional studies on skin fibroblasts generated from patients and HEK293T cells showed that the mutation may impair mitochondrial function and decrease the ability of AFG3L2 protein to enter the mitochondrial inner membrane. In addition, this novel mutation led to protein degradation and reduced the stability of the AFG3L2 protein, which appeared to be associated with the proteasome-ubiquitin pathway.

## Introduction

With a prevalence of 1:12000 to 1:25,000, dominant optic atrophy (DOA) is the most common form of hereditary optic neuropathy [1, 2]. It usually occurs in infancy and presents a prolonged course. The clinical manifestations include moderate to severe vision loss, binocular temporal nerve atrophy, central or paracentric scotoma, and color vision deficiency [3]. Mitochondrial dysfunction and subsequent retinal ganglion cell death are directly associated with visual impairment in DOA [4]. It has been reported that 70–75% of DOA are caused by *OPA1* gene mutations [5]; however, mutations in other genes also cause DOA. The human m-AAA protease, located in the inner membrane of the mitochondria (IMM), is composed of homo-hexamers of AFG3-like protein 2 (AFG3L2) subunits or hetero-hexamers of AFG3L2 subunits and paraplegin (SPG7) subunits [6]. This complex exerts important biological regulatory functions, including protein quality control, protein processing, and mitochondrial protein maturation [7]. AFG3L2, an ATP-dependent protease, is essential for the development of neuron axons [8]. The catalytic domains of AFG3L2 are composed of two subunits, the proteolytic domain, which is responsible for substrate proteolysis, and the ATPase domain. Genetic mutations localized throughout the *AFG3L2* gene are associated with various neurodegenerative disorders [9–11]. Extensive investigations have shown that heterozygous mutations in *AFG3L2* are responsible for autosomal dominant spinocerebellar ataxia type 28 (SCA28) [11], while homozygous mutations in *AFG3L2* are associated with autosomal recessive spastic ataxia syndrome (SPAX5) [12].

Recent studies have shown that DOA is associated with mutations in *AFG3L2* [2, 13, 14]. Unlike the *AFG3L2* mutations that cause SCA28 and SPAX5, which are mainly located in the proteolytic domain, *AFG3L2* mutations that cause DOA are mainly located in the ATPase domain [2]. Pathogenic mutations in the intermembrane space domain (IMSD) have rarely been reported, and consequently, their pathogeny remains largely unknown.

In this study, we present a large family with DOA; we found that a novel *AFG3L2* mutation (c.524T>C, p.F175S) was associated with DOA within the family. Unlike previously reported *AFG3L2* mutations located in or near the ATPase domain, this new mutation was located between the two transmembrane domains (TM) of AFG3L2. Functional studies revealed that F175S mutation impaired mitochondrial function and markedly decreased the stability of the mutant AFG3L2 protein. Mutated AFG3L2 appeared impeded upon entering the IMM and was degraded through a mechanism mediated by the ubiquitin-proteasome pathway.

## Results

### Clinical features

**Fig. 1:**
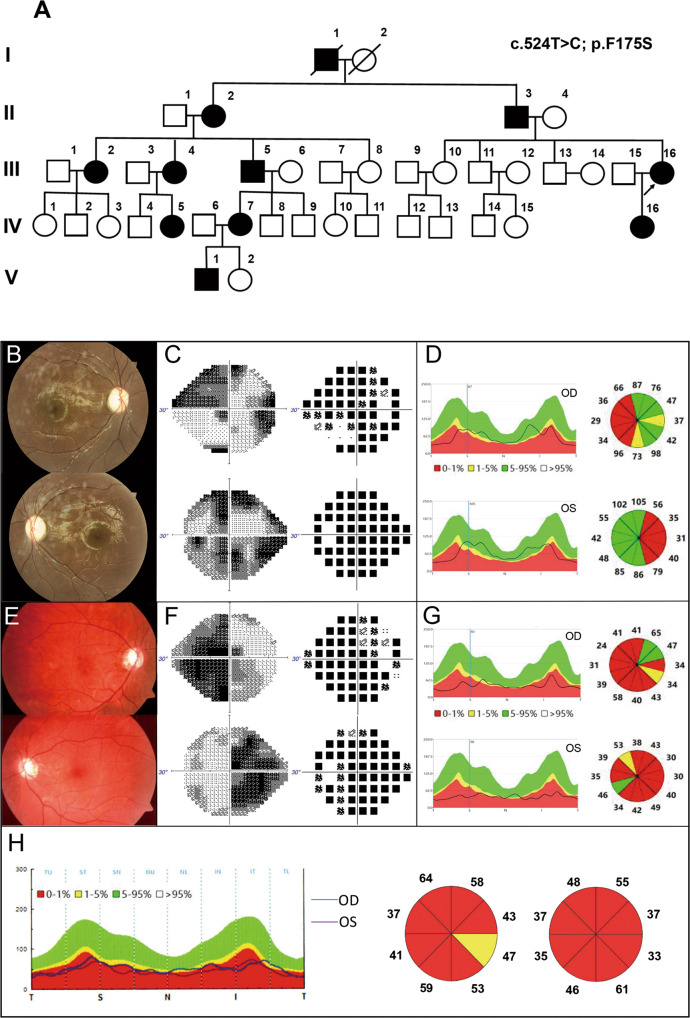
The pedigree and ophthalmologic examinations of 3 representative patients in the family. **A** The Pedigree of the large DOA family. **B**, **E** Fundus photography showed temporal pallor of the optic disc. **C**, **F** Visual field testing presented defect of the visual field. **D**, **G**, **H** SS-OCT exhibited varying degrees of bilateral thinning of RNFL in patients of different ages.

A large five-generation Chinese family with DOA was identified, and the pedigree was shown in Fig. 1A. In this family with 40 individuals, a total of 11 patients were included. The proband (III-16) was a 35-year-old woman. The onset of the disease was when she was 12 with slowly progressive vision loss. On admission, her latest BCVA was 0.5 bilaterally, and color-vision testing revealed abnormal color vision. Visual field testing presented defect of temporal visual field (Fig. 1F). Fundus photography showed temporal pallor of the optic disc (Fig. 1E). SS-OCT exhibited marked bilateral thinning of retinal fiber layer (RNFL) (Fig. 1G). No other extraocular neurological symptoms were observed.

The father of proband (II-3), 75 years of age, presented with reduced vision since childhood. The latest BCVA was 0.04 bilaterally. OCT showed a more severe reduction in RNFL than the proband (Fig. 1H). The daughter of proband (IV-16), a 5-year-old girl, had decrease of vision for years. The latest BCVA was 0.4 bilaterally. Similarly, she manifested abnormal color vision, irregular scotomas in the 30° visual field in both eyes, temporal pallor of the optic disc and bilateral temporal thinning of RNFL (Fig. 1B–D).

Other patients in this family presented similar clinical manifestations to varying degrees (Supplementary Fig. 1). Clinical characteristics of some patients are summarized in Table 1.

**Table 1:** Clinical characteristics of patients with p.F175S (c.524T<C) mutation in this family.

Identifier	Age	Time of onset	VA reduction	BCVA		Constriction of visual field	Abnormal color vision	FO Pallor	OCT (decreased RNFL thickness)
				OD	OS				
II-3	75	childhood	+	0.04	0.04			+	+
III-4	58	childhood	+	0.05	0.03		+	+	+
III-16	35	12	+	0.5	0.5	+	+	+	+
IV-5	15	childhood	+	0.15	0.15		+	+	+
IV-7	35	11	+	0.05	0.1		+	+	+
IV-16	5	childhood	+	0.4	0.4	+	+	+	+
V-1	13	10	+	0.3	0.15		+	+	+

### Genetic analysis revealed a novel missense variant in *AFG3L2*

**Fig. 2:**
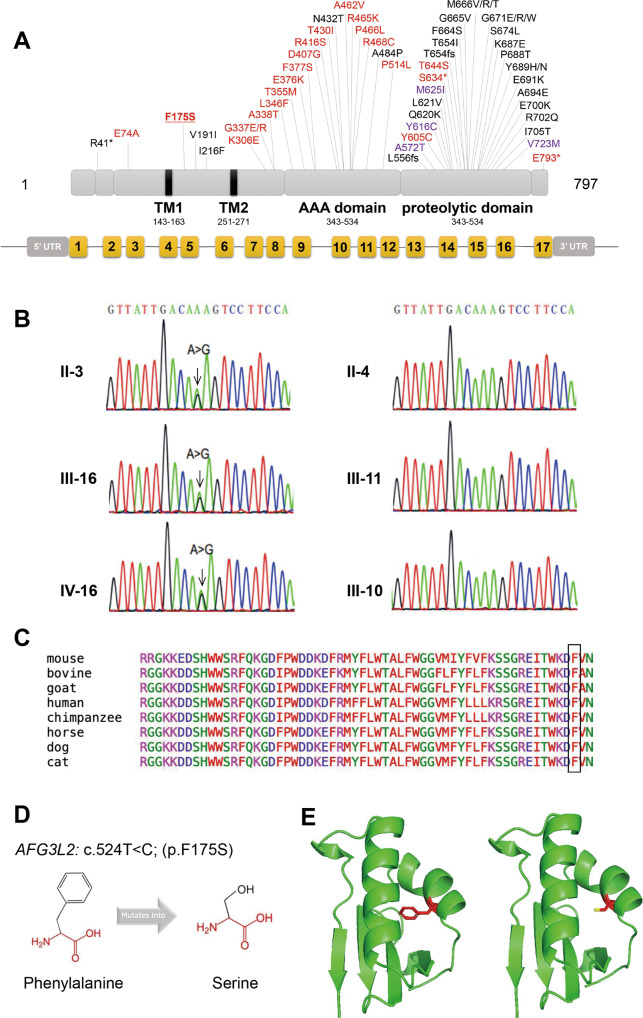
Validation and predictive analysis of the novel mutation. **A** Location distribution of *AFG3L2* mutations related to optic atrophy (red), SCA28 (black), and SPAX5 (purple). The mutation in this study was bold and underlined. (Modified from previous study [2]) **B** Sanger sequencing results in the family numbers. **C** Multiple alignments of Phe175 in AFG3L2 protein among different species. **D** Structural prediction of F175S mutant AFG3L2 protein.

Whole-exome sequencing identified a heterozygous missense mutation c.524T>C (p.F175S), in exon 8 of the *AFG3L2* gene in the proband. This is a novel mutation, not reported in population databases such as 1000 Genomes Project (1000 Genomes), Genome Aggregation Database (GnomAD) or in clinical cases, resulting in serine to phenylalanine acid at position 175 (p.F175S). Interestingly, unlike most previous reports that *AFG3L2*-related DOA variants were predominantly located in the ATPase domain, this novel variant was located between two transmembrane domains (TM1 and TM2), the IMSD of *AFG3L2* (Fig. 2A). Although the key functions of m-AAA protease in proteolysis processing and substrate degradation have been well known, the role of IMSDs remains to be determined. Sanger sequencing of *AFG3L2* revealed segregation of the variant with optic atrophy in participating individuals (Fig. 2B; Supplementary Fig. 2) and followed an autosomal dominant pattern of inheritance. Multiple alignments of amino acid sequences of AFG3L2 protein from different species showed that Phe175 is highly evolutionally conserved among species (Fig. 2C). The in silico tools predicted the variant to be harmful (Table 2). The variant was likely pathogenic according to the ACMG guidelines.

**Table 2:** In silico pathogenicity analyses of F175 variant.

SIFT	Damaging (0.0)
Polyphen-2	Probably damaging (1.0)
LRT	Deleterious (0.000)
Mutation Taster	Disease causing (1)
TATHMM	Damaging (−2.75)
PROVEAN	Damaging (−7.45)
CADD	Damaging (32)

### Protein structure prediction

To predict the effects of p.F175S mutation on protein structure and function, we used the online software HOPE (Fig. 2D). We obtained the structure model of wild-type AFG3L2 protein fragment (2LNA, residues 164-251) [15] containing the amino acid residue we studied with the Swiss-Model. We further predicted the protein structure model of F175S mutant AFG3L2 by the PyMOL software (Fig. 2E). A Phenylalanine mutated to a Serine at position 175 located on the Alpha 1 helix. Compared with the wild-type residue, the mutant residue was smaller and more hydrophilic.

### F175S mutation induced mitochondrial dysfunction

We tested the effect of mutation on mitochondrial function in two human cell lines in vitro. The mitochondrion is the major source of reactive oxygen species (ROS) production [16]. Mitochondrial dysfunction causes accumulation of ROS, which impairs the function and survival of neurons [17]. Besides, hyper-levels of ROS enhance the dissipation of MMP, which is a characteristic of mitochondrial damage at the early stage [18]. Previous studies have found that mutations in DOA may lead to an increase in ROS and a decrease in MMP [19, 20].

**Fig. 3:**
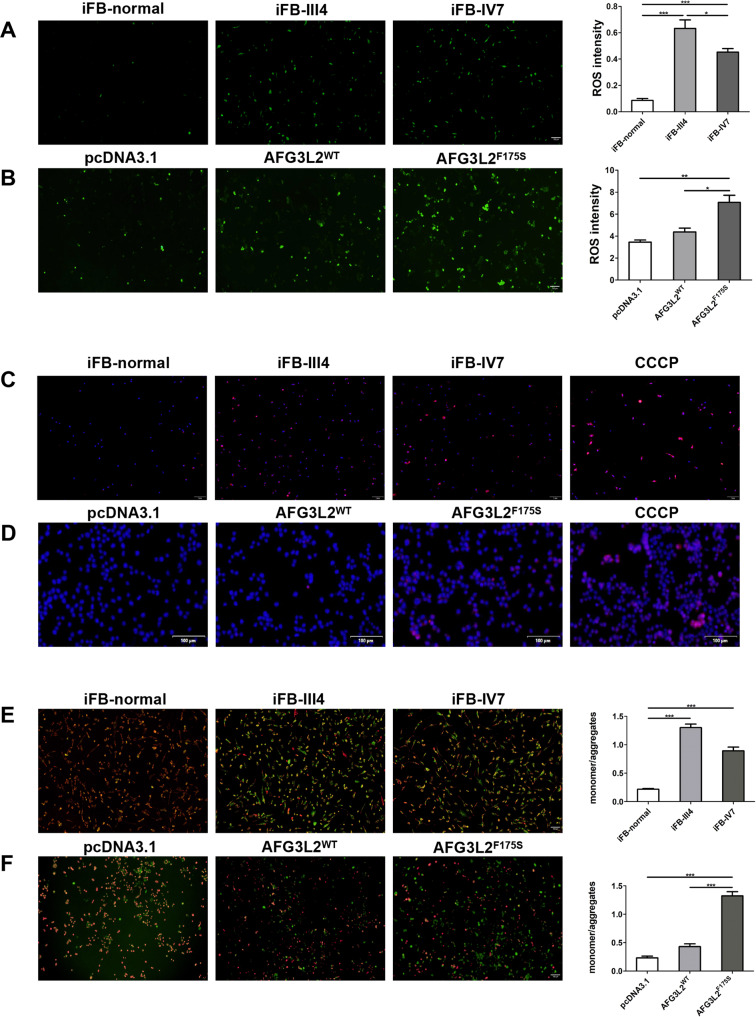
F175S mutation caused mitochondrial damage. **A**, **B** The cytoplasmic ROS staining (green) in human normal and F175S AFG3L2 mutation iFB cells and HEK293T cells. Cell density was shown in Supplementary Fig. 3. **C**, **D** The mitochondrial ROS staining (red) in human normal and F175S AFG3L2 mutation iFB cells and HEK293T cells. Cells treated with CCCP were used as positive control. **E**, **F** MMP in wild-type and F175S AFG3L2 mutation iFB cells and HEK293T cells. Green fluorescence and red fluorescence represent JC-1 monomer and JC-1 aggregates, respectively. Cell transfection efficiency was shown in Supplementary Fig. 4. (Values expressed as mean ± SD, *p < 0.05, **p < 0.01, ***p < 0.001).

We generated immortalized skin fibroblasts from a healthy person (III-8) and two DOA patients (III-4, IV-7) in this family. DCFH-DA and Mito-SOX were used for the detection of cytoplasmic and mitochondrial ROS, respectively. JC-1 assay kit was used for the MMP analysis. Compared with the healthy control, both cytoplasmic (Fig. 3A) and mitochondrial (Fig. 3C) ROS in the fibroblasts of DOA patients were significantly increased, and the ratio of JC-1 monomer to aggregates increased (Fig. 3E), indicating MMP decreased in the patients.

To further verify the mitochondrial dysfunction caused by the novel mutation, we constructed wild-type (*AFG3L2*^WT^) and mutant (*AFG3L2*^*F175S*^) *AFG3L2* plasmids using pcDNA3.1 as vectors and transfected them into HEK293T cells. 24 hours after transfection with 2 µg of plasmid into each well of cells cultured on six-well plates, ROS levels were significantly increased and MMP was markedly decreased in the *AFG3L2*^*F175S*^ group when compared with the no-load (pcDNA3.1) and *AFG3L2*^*WT*^ groups (Fig. 3B, D, F). This was inconsistent with the results seen in the immortalized fibroblasts.

**Fig. 4:**
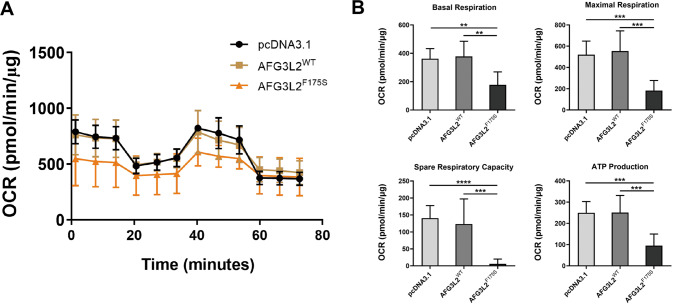
F175S mutation affected the mitochondrial function. **A** Average seahorse profiles demonstrating oxygen consumption rate (OCR). **B** Representative bar graphs demonstrating basal respiration, maximal respiration, spare respiratory capacity and ATP production. (Values expressed as mean ± SD, pcDNA3.1: *n* = 7, AFG3L2^WT^: *n* = 5, AFG3L2^F175S^: *n* = 5, **p < 0.01, ***p < 0.001, ****p < 0.0001).

In the following investigation the influence of F175S mutation on mitochondrial metabolism via Seahorse XFe Analyzer (Fig. 4). In Mito Stress Test (Fig. 4A), basal respiration, maximal respiration, spare respiratory capacity, and ATP production (Fig. 4B) were all reduced in mitochondrial metabolism in the *AFG3L2*^*F175S*^ group. There is no marked difference between no-load group and *AFG3L2*^*WT*^ group.

### F175S mutation damaged the stability of AFG3L2 protein through the ubiquitination-proteasome pathway

It has been found that expression levels of the construct consisting only of the ATPase and peptidase domains of human AFG3L2 (residues 272–797), abandoning the N-terminal transmembrane regions, dropped rapidly under continual induction in E. coli [6, 7]. Therefore, we hypothesized that the N-terminal transmembrane domain might be involved in maintaining protein stability. AFG3L2 encodes a subunit of the m-AAA, which is responsible for protein quality control in IMM. The decreased stability of AFG3L2 protein might impair the normal function of mitochondria.

**Fig. 5:**
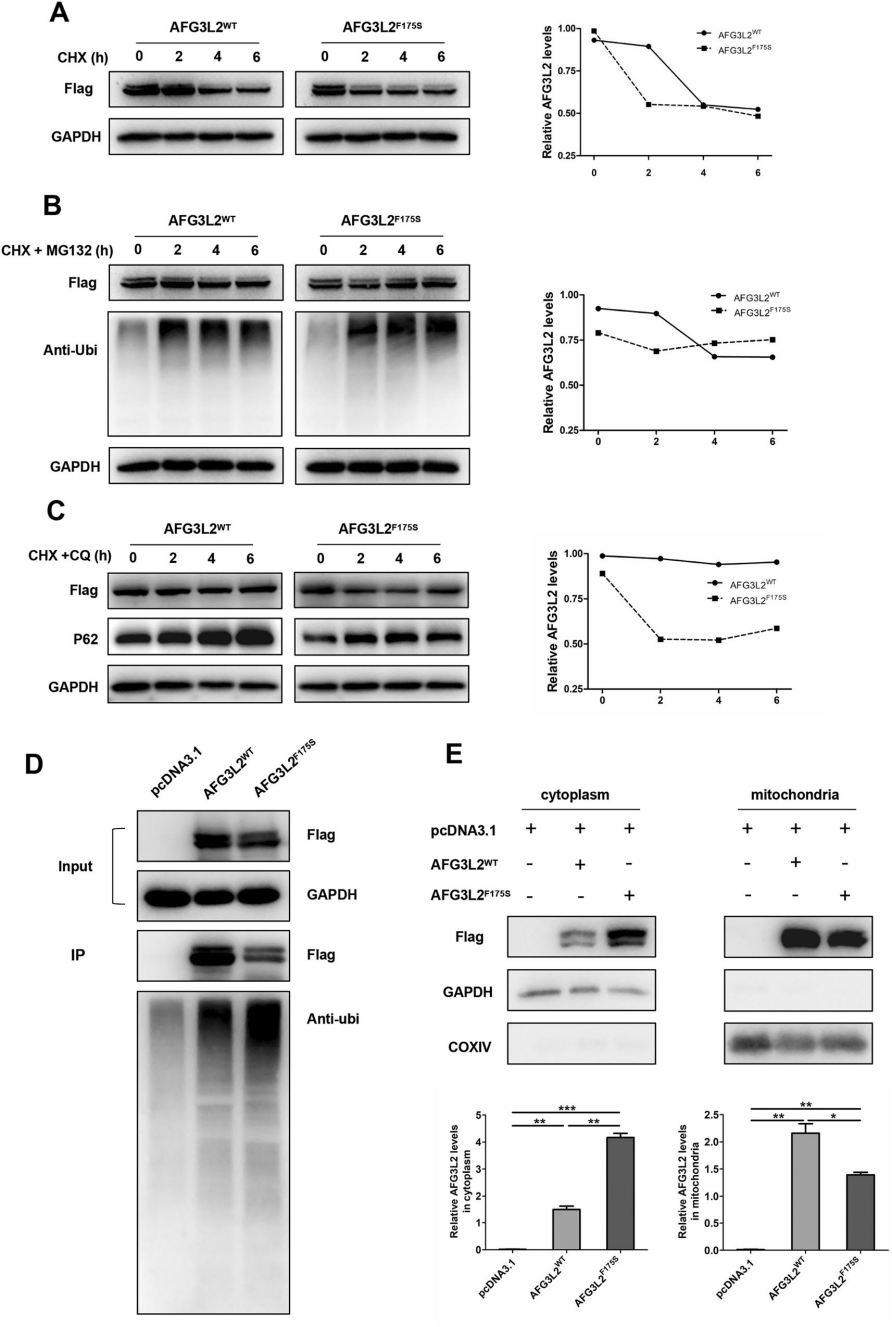
F175S mutation damaged the stability of AFG3L2 protein may through the ubiquitination-proteasome pathway. **A** HEK293T cells were transfected with wild type and F175S mutant plasmids for 24 h, and treated with 100 μM CHX, the expression of AFG3L2 protein was detected. **B, C** AFG3L2 protein stability was detected after cells were treated with 50 μM MG132 and 20 μM chloroquine, respectively. **D** 24 h after transfection, wild type and mutant AFG3L2 proteins were enriched by immunoprecipitation, and their ubiquitination levels were detected. **E** Wild and mutant AFG3L2 proteins in mitochondria and cytoplasm. (Values expressed as mean ± SD, *p < 0.05, **p < 0.01, ***p < 0.001).

To investigate if the mutation of AFG3L2 affect the protein’s stability, we treated each group of HEK293T cells with 100 μM CHX for 0, 2, 4, and 6 h after plasmid transfection, respectively. Compared with wild-type AFG3L2 protein, the half-life of AFG3L2 protein carrying F175S variant was significantly shortened (Fig. 5A), suggesting that F175S mutation impaired the stability of AFG3L2 protein.

The ubiquitin-proteasome system (UPS) and the autophagosomal-lysosomal pathway (ALP) are the two main protein degradation routes of eukaryotic cells. To further explore the pathway associated with protein degradation, we treated the transfected cells with inhibitors of proteasome (MG132) and lysosomal enzymes (chloroquine) when adding CHX. As shown in Fig. 5 B and C, chloroquine significantly prolonged the half-life of AFG3L2^WT^ protein, while MG132 only slightly prolonged the protein half-life. On the contrary, the half-life of AFG3L2^F175S^ protein was not obviously improved by chloroquine, but prolonged after MG132 treatment. Therefore, it can be interpreted that both proteasome and lysosomal enzymes were involved in the degradation of wild-type AFG3L2 protein, while the lysosomal pathway may be the main degradation pathway. However, the F175S mutant AFG3L2 protein may be mainly degraded by proteasome.

Subsequently, we examined the ubiquitination levels of AFG3L2 protein in cells transfected with different plasmids and found that the ubiquitination level of the AFG3L2^F175S^ protein increased visibly compared with the wild-type AFG3L2 protein (Fig. 5D). This further confirmed that the reduced stability of the F175S mutant protein may be mainly mediated by the ubiquitin–proteasome pathway.

### F175S mutation reduced the level of AFG3L2 protein in the mitochondria

Considering that proteasomes predominantly exist in cytoplasm and primarily degrade cytoplasmic proteins [21], we speculated that the AFG3L2^F175S^ protein mainly exists in the cytoplasm rather than in the mitochondria. Mitochondrial proteins and cytoplasmic proteins were extracted respectively 24 h after plasmids transfection. We determined the levels of AFG3L2 protein in the mitochondria and cytoplasm, and found that compared with that of wild-type protein, the expression of AFG3L2^F175S^ protein decreased in the mitochondria and increased in the cytoplasm (Fig. 5E). We determined the mitochondrial localization of AFG3L2 protein using immunofluorescence microscopy. While almost all the wild-type AFG3L2 co-localized with the mitochondria, there was a small amount of mutant AFG3L2 did not co-localize with the mitochondria (Supplementary Fig. 5). Therefore, it can be inferred that the AFG3L2 proteins carrying F175S mutation may be impeded from localizing to the mitochondria or across the mitochondrial membranes. The F175S mutation was located between the two transmembrane domains at the N-terminus of AFG3L2. When the 175th residue mutated from phenylalanine to serine, hydrophilicity was enhanced, impeding the translocation of the mutant into the IMM. Therefore, the mutant protein may accumulate in the cytoplasm and be degraded by the ubiquitin–proteasome pathway.

## Discussion

*AFG3L2* mutations can lead to SPAX5 and SCA28 by impairing oxidative phosphorylation and mitochondrial calcium homeostasis in Purkinje neurons of the cerebellum [9, 12, 22]. Recent studies on *AFG3L2* have highlighted the pathogenic role of AFG3L2 in *OPA1* mutation-negative DOA, which attracted our attention. The novel *AFG3L2* missense mutation (c.524T>C, p.F175S) has never been reported in human databases or in the literature. More interestingly, the novel mutation was not in the ATPase domain, the hot mutation region leads to DOA, instead between the two transmembrane domains of AFG3L2. Functional studies conducted on p.F175S mutation demonstrated its pathogenicity, which may be attributed to reduced stability of AFG3L2 protein mediated by the ubiquitin-proteasome pathway.

With no other extraocular neurological symptoms, the novel *AFG3L2* mutation (c.524T>C, p.F175S) caused isolated DOA. Similar to patients with DOA caused by mutations in the ATPase domain of *AFG3L2* and other genes, our patients also developed the disease in childhood, presenting with decreased visual acuity, impaired visual field, color vision disorder, pale optic disc, and thinning of RNFL. Moreover, disease severity increased with age (Fig. 1 B–H).

Protein quality control is essential for cellular function and survival in all organisms [23]. *AFG3L2* and *SPG7* encode the cognate subunits of the mitochondrial m-AAA protease complex [24, 25], which perform protein quality control by degrading misfolded, damaged, and non-assembled subunits of the IMM [26, 27]. Our data showed that F175S mutation may reduce the level of AFG3L2 in the mitochondria. In addition, the F175S mutant AFG3L2 protein degraded more rapidly and was less stable than the wild-type protein, possibly causing insufficient protein levels of AFG3L2 for normal protein quality control in the IMM. Consequently, the accumulation of misfolded, damaged, and non-assembled proteins may occur in the mitochondria, resulting in mitochondrial dysfunction, such as increased production of ROS and MMP imbalance.

UPS and ALP are the two main protein degradation routes in eukaryotic cells and are crucial for proteostasis [28]. UPS is mainly responsible for the degradation of unneeded or misfolded proteins, while ALP is responsible for the bulk degradation of organelles and long-lived proteins [29]. We found that both proteasomal and lysosomal degradation pathways were involved in the degradation of the wild-type proteins, but lysosomal pathway was the dominant pathway. However, the F175S AFG3L2 mutant proteins may be degraded mainly by ubiquitin–proteasome pathway. On the one hand, this may be linked to structural changes in the protein arising from F175S mutation, as one of the major functions of ubiquitin is the targeting of misfolded proteins. On the other hand, it may be partly related to the difference in the locations of wild-type and mutant AFG3L2 proteins because proteasomes predominantly exist in the cytoplasm and primarily degrade cytoplasmic proteins.

Our results showed that the mutant protein could also enter the mitochondria, but compared with that of the wild-type protein, the level of F175S AFG3L2 mutant protein in the mitochondria reduced, whereas that in the cytoplasm increased. This indicated that AFG3L2 carrying the F175S mutant may have a transmembrane barrier. This may be related to the location of the point mutation, which is close to the N-terminus. The AFG3L2 protein has a signal peptide at the N terminal. In addition, the F175S mutation was located between the two transmembrane domains. When the 175th residue mutated from phenylalanine to serine, hydrophilicity was enhanced, and this possibly impeded the translocation of the mutant into the IMM.

Mutations in the *OPA1* gene are the leading cause of DOA. OPA1 is a dynamin-like GTPase protein necessary for mitochondrial fusion [30], maintenance of inner membrane integrity, and regulation of cell apoptosis [31]. Previous studies have shown that in AFG3L2-related DOA, point mutations in the ATPase domain of AFG3L2 disrupt the stability of the OPA1 long isoform, leading to mitochondrial fragmentation [2, 14]. We identified a novel F175S mutation in a large DOA family, and this mutation may reduce the stability of AFG3L2 protein. Further studies are necessary to clarify the relationship between the novel AFG3L2 mutation and OPA1 processing.

In summary, this study is, to the best of our knowledge, the first to demonstrate that isolated DOA is caused by an *AFG3L2* mutation located between two transmembrane domains and to explore its pathogenesis. We presented the case of a large DOA family with a novel *AFG3L2* mutation (c.524T>C, p.F175S) located in the intermembrane space domain. Unlike previous reports of DOA causing *AFG3L2* mutations that are predominantly located in or near the ATPase domain, this novel mutation was located between the two transmembrane domains of AFG3L2. In addition to in silico analysis, functional studies on the mutation further demonstrated its pathogenicity by providing evidence that it damaged mitochondrial function. The capacity of the F175S mutant AFG3L2 protein to localize to the mitochondria was impaired and mitochondrial protein levels were reduced. In addition, compared with that of the wild-type AFG3L2 protein, the stability of the mutant protein was markedly decreased, and the reduction was seemingly associated with the ubiquitin-proteasome pathway. Thus, our work widens the repertoire of pathogenic mutations associated with DOA.

## Materials and methods

### Genetic screening

After informed consent was obtained, whole blood was collected for whole exome Sequencing (WES). Genomic DNA was extracted from blood with the TIANGEN Blood DNA Kit (DP304, TIANGEN, China). Whole Exome Capture AIExomeV2 kit (iGeneTech Co., Beijing, China) was used for WES. The whole exon region was enriched using a liquid phase probe method and sequenced on the Illumina Nova sequencing platform (Illumina, Inc., California, USA) according to the manufacturer’s standard operating protocol. A depth of 100× was ensured in sequencing and the coverage of targeted exons was no less than 99%. Sequence data were uploaded to NCBI Sequence Read Archive with accession number SRR19049154.

### In silico analysis

The variant was classified according to ACMG guidelines. SIFT, Polyphen-2, Mutation Taster, PROVEAN, FATHMM, CADD, and LRT were used to predict the pathogenicity of the mutation. Clustal Omega was applied to align the protein sequence among various species. Swiss-Model, PyMOL, and HOPE were used to predict the effects of the mutation on protein structure and function [32, 33].

### Antibodies and reagents

Antibodies and reagents were purchased from: mouse anti-flag (F1804, Sigma, USA); rabbit anti-flag (ab205606, Abcam, USA); rabbit anti-AFG3L2 (14631-1-AP, Proteintech, USA); rabbit anti-ubiquitin (ET-1609-21, HUABIO, China); mouse anti-COXIV (11967S, Cell Signaling Technology, USA); rabbit anti-vimentin (5741, Cell Signaling Technology, USA); rabbit anti-GAPDH (10494-1-AP, Proteintech, USA); anti-flag magnetic beads (HY-K0207, MCE, USA); cycloheximide (CHX, 66-81-9, Selleck, USA); MG132 (HY-13259, MCE, USA); chloroquine (CQ, HY-17589A, MCE, USA).

### Cell culture

HEK293T cell line was purchased from American Type Culture Collection (ATCC, Manassas, VA, USA) and identified by STR analysis. Cells were maintained in high glucose DMEM supplemented with 10% FBS and 100 units of penicillin/streptomycin (32105, Mengbio, China) at 37 °C with 5% CO_2_.

Skin fibroblasts were derived from one healthy person (III-8) and two DOA patients (P1: III-4 and P2: IV-7) from the family. Fibroblasts were cultured in DMEM medium supplemented with 10% FBS, 100 units of penicillin/streptomycin at 37 °C with 5% CO_2_. The specific vimentin antibody was used to identify the cells (Supplementary Fig. 6). All cell lines were tested for mycoplasma contamination by using a commercially available kit (AC16L061, Shanghai Life-iLab Biotech, China)

### Plasmids and transfection

The CDS sequences of wild-type AFG3L2 (NM_006796.3) and c.524T>C (p.F175S) containing a C-terminal Flag tag were synthesized cloned into the pcDNA3.1 vector, respectively. All constructs were verified through Sanger sequencing (Sangon Biotech, China). Cells were transfected with Lipofectamine 3000 (L3000015, Invitrogen, USA) and 2 μg of DNA construct (per 3 × 10^5^ cells) according to the manufacturer’s instructions.

### ROS assays

The cytoplasmic ROS level was detected by measuring the oxidative conversion of cell-permeable dichlorodihydrofluorescein diacetate (DCFH-DA; S0033; Beyotime, China) to green fluorescent 2′,7′-dichlorofluorescein. The experimental operation was carried out according to the instructions. DCFH-DA was diluted with serum-free medium at 1:1000 to a final concentration of 10 µM. Cells were cultured in 6-well plates, and 1 ml of diluted DCFH-DA was added to each well, and incubated in a 37 °C incubator for 20 min. Then, cells were washed three times in serum-free culture medium. Visualized the cells with fluorescence microscopy (Ex/Em = 488/525 nm). Images were analyzed using the ImageJ software (Version 1.52a, NIH).

The mitochondrial ROS was measured by MitoSOX^TM^ Red reagent (M36008, Invitrogen, USA) following the experimental protocol. The cells were incubated with 5 µM MitoSOX^TM^ Red reagent working solution diluted with HBSS for 10 min at 37 °C. Then wash cells gently three times with warm buffer. The nucleus was stained with Hochest. Visualized the cells with fluorescence microscopy.

### JC-1 assays

JC-1 assay kit (C2006; Beyotime, China) was used for the mitochondrial membrane potential (MMP) analysis. Cells cultured in 6-well plates. After the medium was removed and the cells were washed with PBS, 1 ml of medium and 1 ml of JC-1 working solution were added, then the cells were incubated in the 37 °C cell incubator for 20 min. After incubation, supernatant was removed, and cells were washed twice with JC-1 staining buffer. Fluorescence microscopy was used to analysis the change in MMP (Δψm), which was expressed as the ratio of green fluorescence to red fluorescence (ratio of JC-1 monomers/JC-1 aggregates). Images were analyzed using the ImageJ software (Version 1.52a, NIH).

### Cell metabolic assays

The oxygen consumption rate (OCR), reflecting the function of respiratory chain, was measured by XFe analyzer (XFe96, Agilent Seahorse Technologies, USA). The HEK293T cells were seeded in XF 96-well (102601-100, Agilent Seahorse Technologies, USA) at 5 × 10^3^/well and allowed to adhere overnight. The XF Cell Mito Stress Test kit (103015-100, Agilent Seahorse Technologies, USA) was used following the manufacturer’s instruction to detect the mitochondrial metabolism of the cells transfected with plasmids for 24 h. 15 μM oligomycin, 5 μM FCCP, and 5 μM rotenone/antimycin-A were injected sequentially in probe plate during detection. The cells were then observed under the microscope, and the wells with a low number of cells were excluded. Finally, Protein concentration was measured in each well for normalization. The software Wave 2.6.3 was used for results analysis.

### Western blotting

Total protein was extracted by RIPA lysate containing 1% cocktail and mitochondrial protein was extracted by the mitochondrial protein extract kit (C3601, Beyotime, China). Protein was separated in sodium dodecyl sulfate-polyacrylamide gel electrophoresis system. Then the protein was transferred onto a PVDF membrane (IPVH00010, Millipore, USA). After blocking in 5% milk for 2 h, the membranes incubated the primary antibody at 4 °C overnight. Then membranes were incubated with second antibody-HRP conjugate for 2 h at room temperature and visualized with Chemiluminescent detection reagent (WBKLS0500, Millipore, USA). Bands were analyzed using the ImageJ software (Version 1.52a, NIH).

### Immunofluorescence

The cells were fixed in 4% paraformaldehyde for 10 min, followed by washing in PBS, permeabilized with 0.1% Triton-100 for 10 min and blocked in 5% FBS for 30 min. Then incubated cells with anti-flag (1: 200; ab205606, Abcam, USA) and anti-COXIV (1: 100; 11967S, Cell Signaling Technology, USA) antibodies overnight at 4 °C. After being washed with PBS three times, secondary anti-mouse Alexaflor 594 (1:250; abs20017, Absin, China) and anti-rabbit IgG-FITC antibodies (1:250; abs20004, Absin, China) were then applied for 2 h at room temperature. Nuclei were stained with DAPI. Zeiss confocal microscope (Zeiss NLO780; Zeiss, Germany) was used to obtain fluorescence microscopy images.

### Immunoprecipitation

HEK293T cells cultured in 10 cm dishes were transfected with 8 μg pcDNA3.1 empty vector, flag-AFG3L2^WT^ and flag-AFG3L2^F175S^ plasmids for 24 h and treated with 50 µM MG132 for 6 h, respectively. Protein lysates were extracted by immunoprecipitation buffer (BL509A, Biosharp, China) containing 1% cocktail. 200 µg proteins and a quarter of protein volume of 5× SDS were mixed to prepare the input sample. 1.5 mg proteins were incubated with 10 µg anti-Flag magnetic beads (HY-K0207, MCE, USA) for 4 h at 4 °C on a slowly moving rotor. Then, the beads were collected by Magnetic Separator and washed by immunoprecipitation buffer containing 1% cocktail three times. The beads were mixed with 1× SDS and boiled for 5 min. The supernatant was collected and analyzed by Western blotting.

### Statistical analysis

The experimental data were analyzed by GraphPad Prism 8 software (GraphPad Software, Inc., USA). One-way ANOVA followed by a Bonferroni correction were devoted to multiple comparisons. All experiments were performed at least two or three independent experiments. The number of each sample was more than 3 (*n* ≥ 3). The *P* < 0.05 was seen as significantly different.

## Supplementary information

Supplementary Figure Legends

Supplementary figure 1

Supplementary figure 2

Supplementary figure 3

Supplementary figure 4

Supplementary figure 5

Supplementary figure 6

STR analysis of HEK293T

Original Data File

## Data Availability

All data generated or analyzed during this study are included in this published article and its supplementary information files.
