# Supplementary material for: A novel mutation located in the intermembrane space domain of AFG3L2 causes dominant optic atrophy through decreasing the stability of the encoded protein
Source: Cell Death Discov. 2022 Aug 15;8:361. doi: 10.1038/s41420-022-01160-9 (PMC9378676; doi:10.1038/s41420-022-01160-9)
Supplement: Supplementary file 1 — Supplementary Figure Legends [file 41420_2022_1160_MOESM1_ESM.docx]

**Supplementary Figure Legends:**

**Supplementary figure 1: Fundus photography and OCT examination of III-8, IV-7, V-1, III-4, and IV-5 in this family.** The fundus examination of III-8 showed no abnormality and the thickness of RNFL was normal. Other DOA patients showed varying degrees of temporal optic disc pallor and thinning of the RNFL. (III-4 had severe nystagmus in her right eye and failed to undergo OCT examination. IV-5 had severe nystagmus in both eyes, and OCT examination could not be performed.)

**Supplementary figure 2: Sanger sequencing of family numbers III-8, IV-7, V-1, III-4, and IV-5.** III-8 did not carry the c.524T>C, p.F175S mutation in *AFG3L2* gene, others carried the mutation.

**Supplementary figure 3: Bright field images of fibroblasts and HEK293T cells in DCFH-DA experiment.**

**Supplementary figure 4: Cell transfection efficiency in DCFH-DA, Mito-SOX and JC-1 experiments. A.** Cell transfection efficiency in DCFH-DA experiment. **B.** Cell transfection efficiency in Mito-SOX experiment. **C.** Cell transfection efficiency in JC-1 experiment.

**Supplementary figure 5:** **The capability of the mutant protein entering the mitochondrial decreased.** COXIV and flag were applied to mark mitochondria and transfected AFG3L2 protein, respectively. While almost all the wild-type AFG3L2 co-localized with mitochondria, there was mutant AFG3L2 that did not co-localize with mitochondria (green in the merged figure). Scale bar 20 μm, magnification × 400.

**Supplementary figure 6: Identification of normal and patients’ fibroblasts.** The vimentin, a typical fibroblast marker, was localized in the cytoplasm of the fibroblasts. The scale bar 100 μm, magnification ×200.
