# Supplementary figures and images for: A novel mutation located in the intermembrane space domain of AFG3L2 causes dominant optic atrophy through decreasing the stability of the encoded protein
Source: Cell Death Discov. 2022 Aug 15;8:361. doi: 10.1038/s41420-022-01160-9 (PMC9378676; doi:10.1038/s41420-022-01160-9)

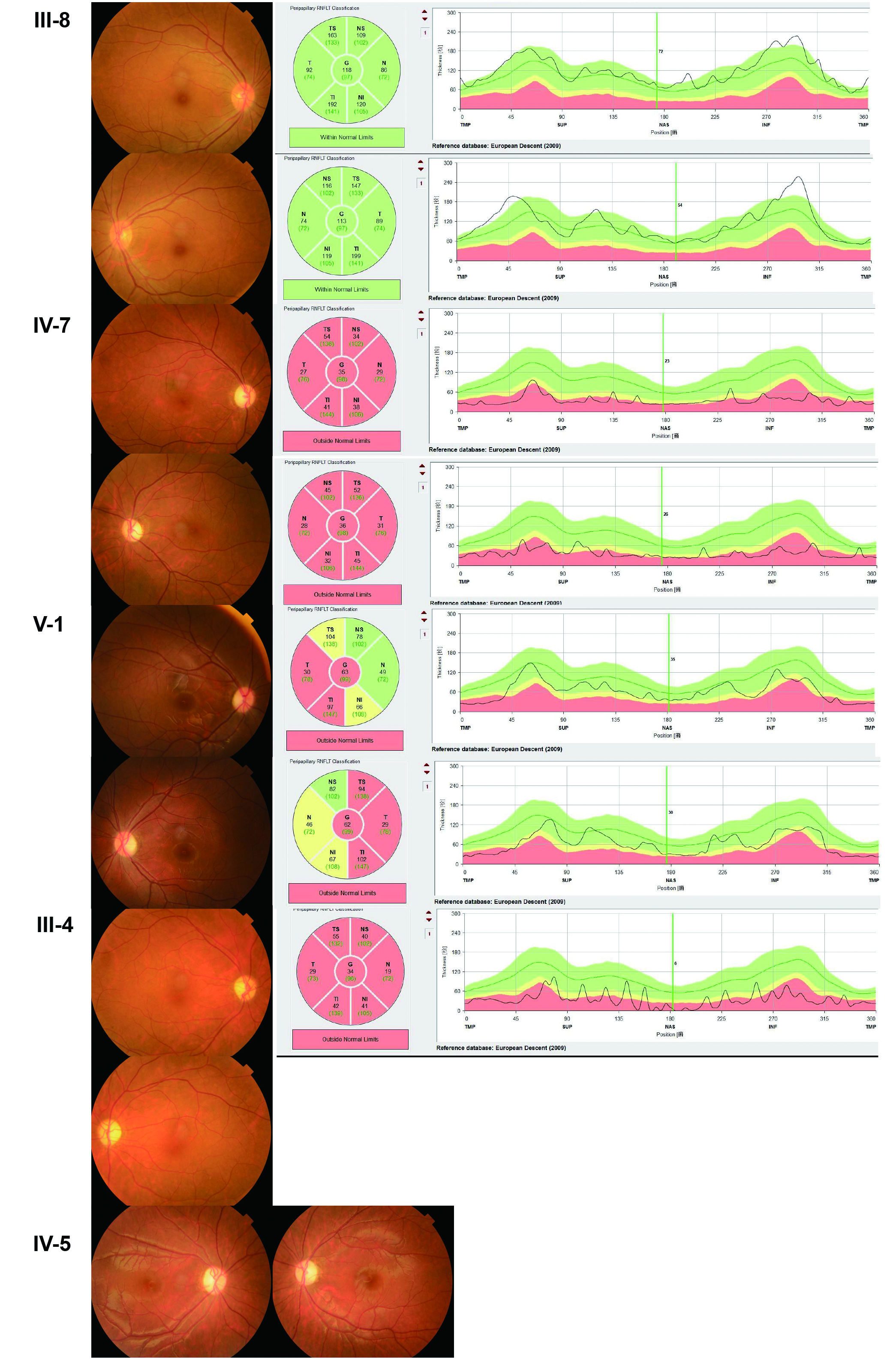

Supplement: Supplementary file 2 — Supplementary figure 1 [file 41420_2022_1160_MOESM2_ESM.tif]

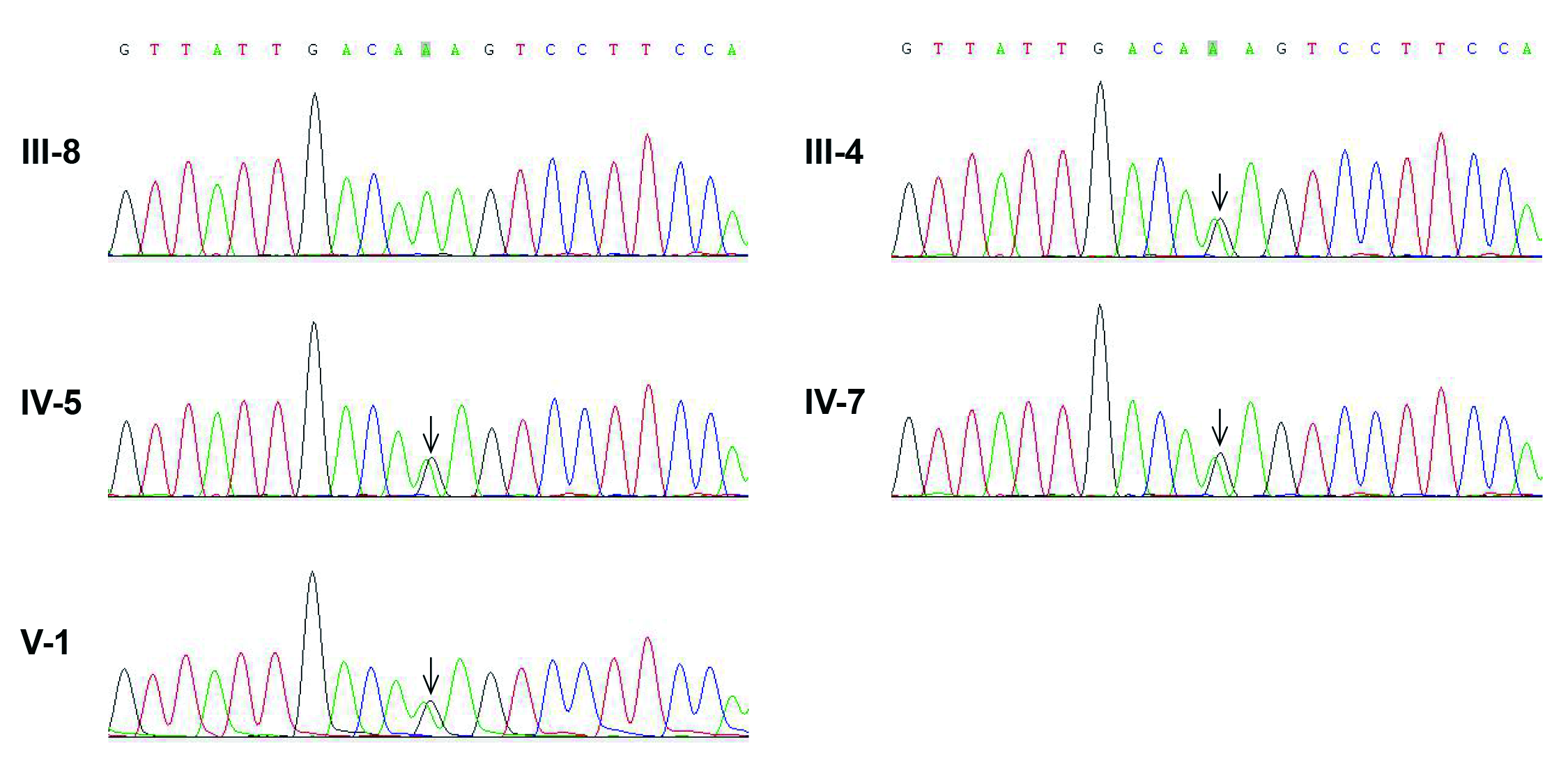

Supplement: Supplementary file 3 — Supplementary figure 2 [file 41420_2022_1160_MOESM3_ESM.tif]

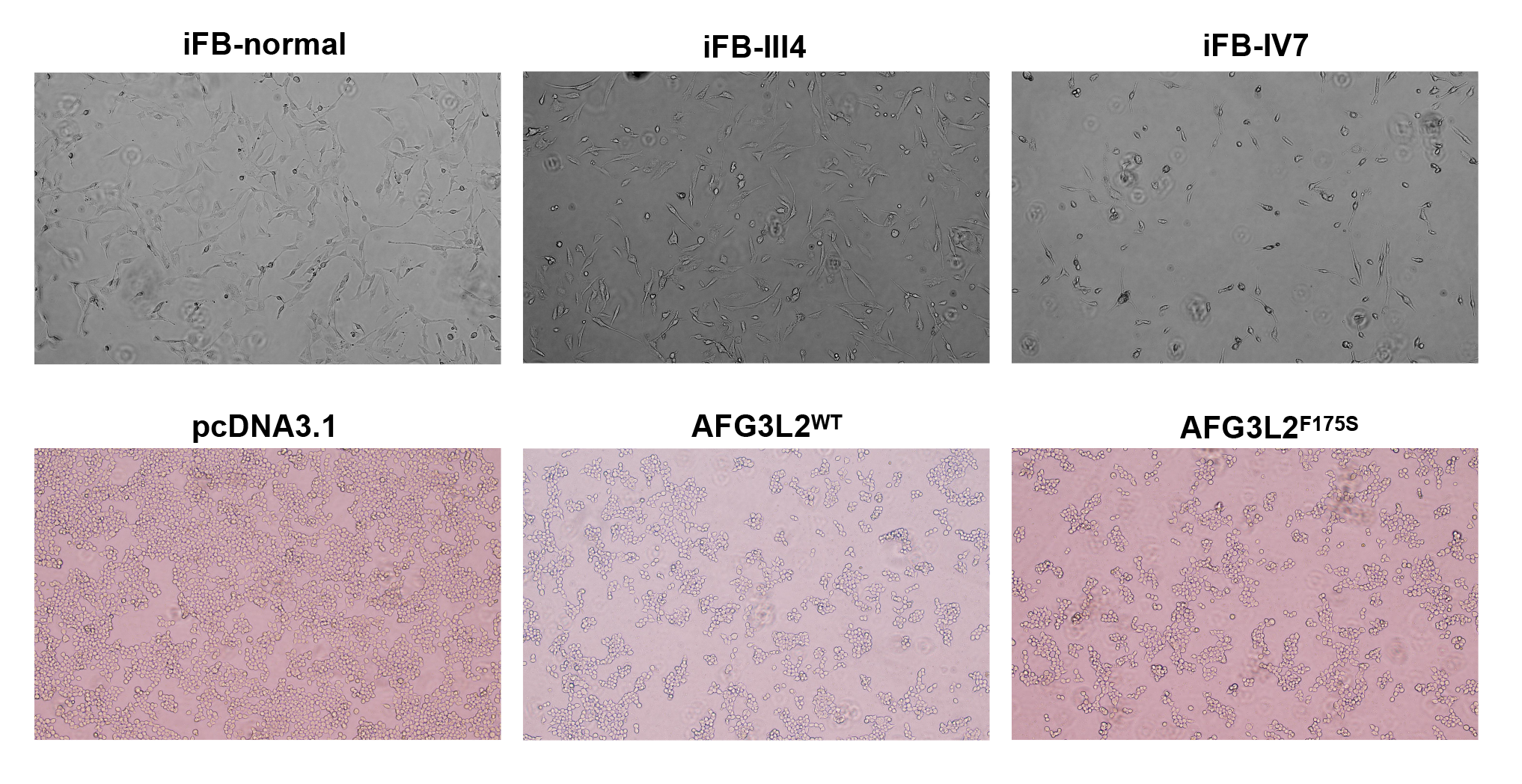

Supplement: Supplementary file 4 — Supplementary figure 3 [file 41420_2022_1160_MOESM4_ESM.tif]

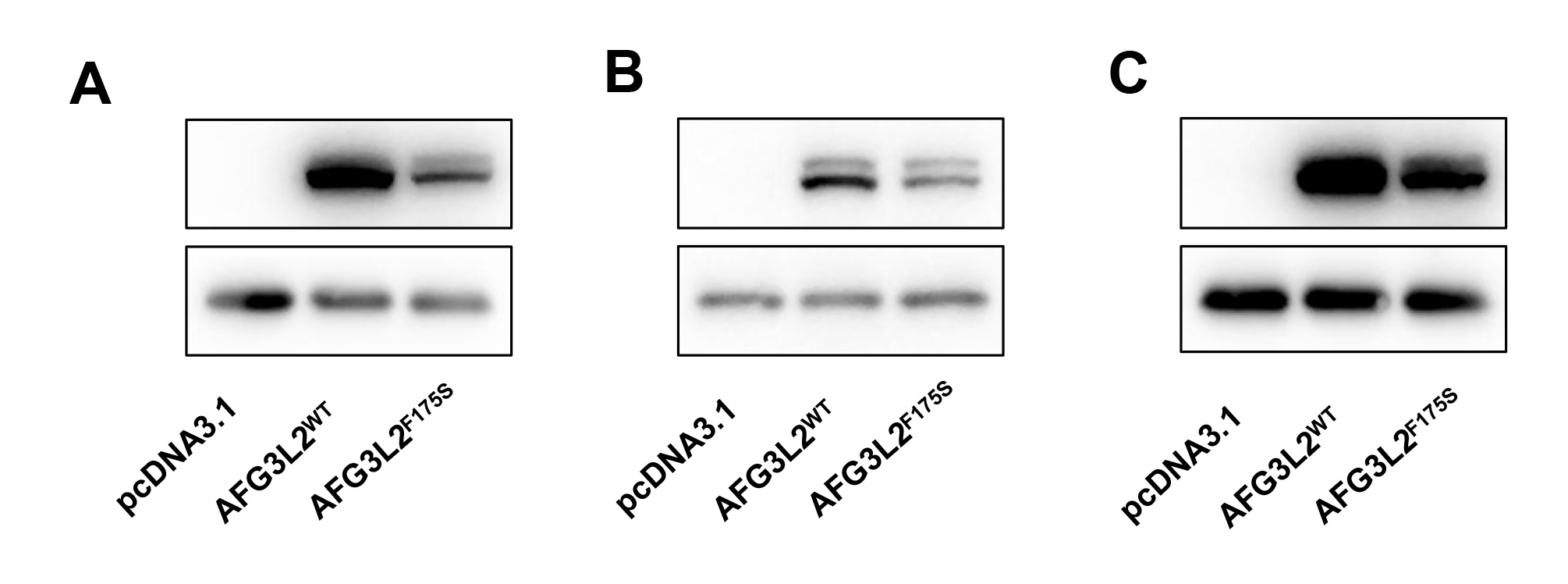

Supplement: Supplementary file 5 — Supplementary figure 4 [file 41420_2022_1160_MOESM5_ESM.tif]

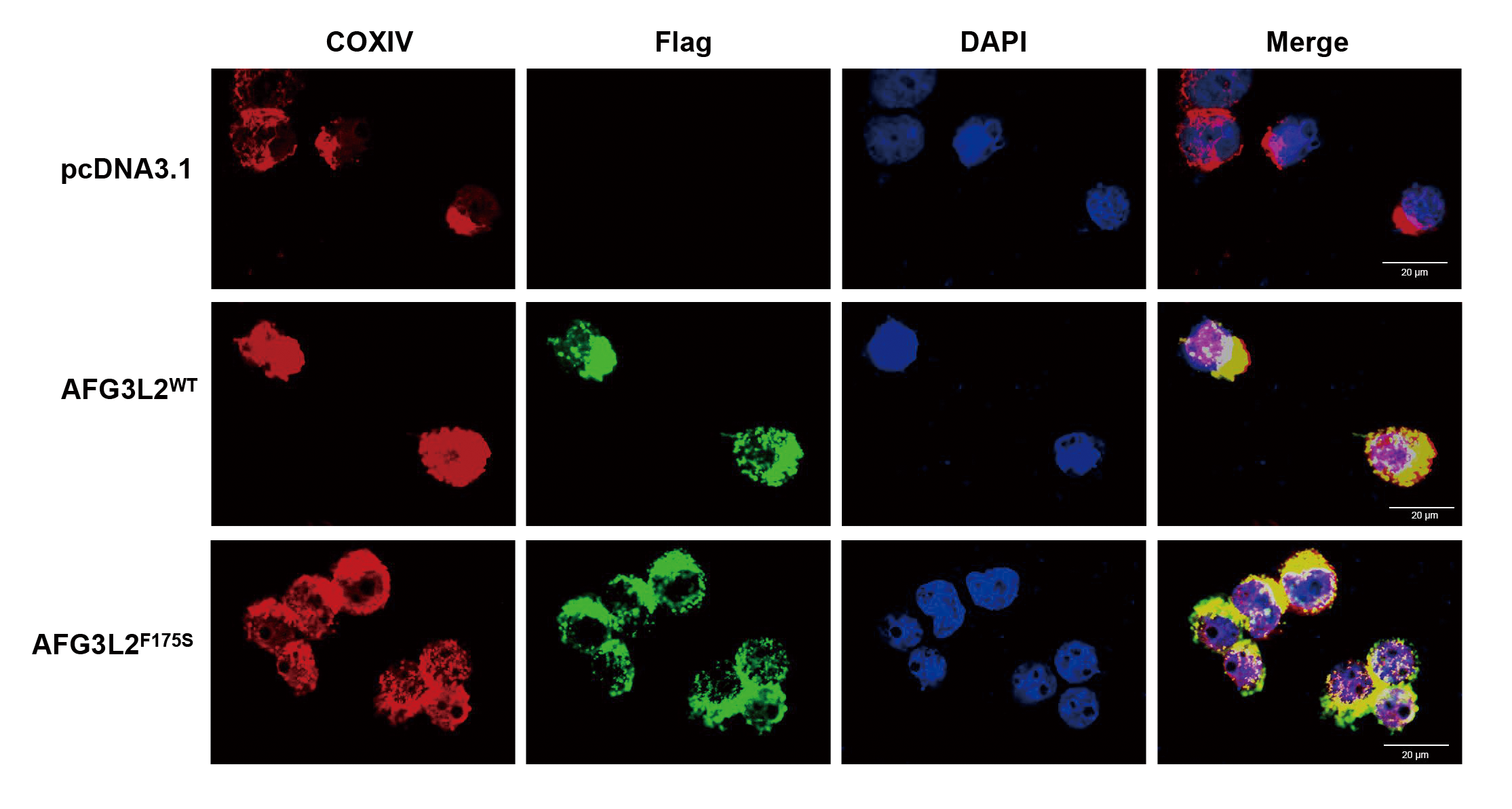

Supplement: Supplementary file 6 — Supplementary figure 5 [file 41420_2022_1160_MOESM6_ESM.tif]

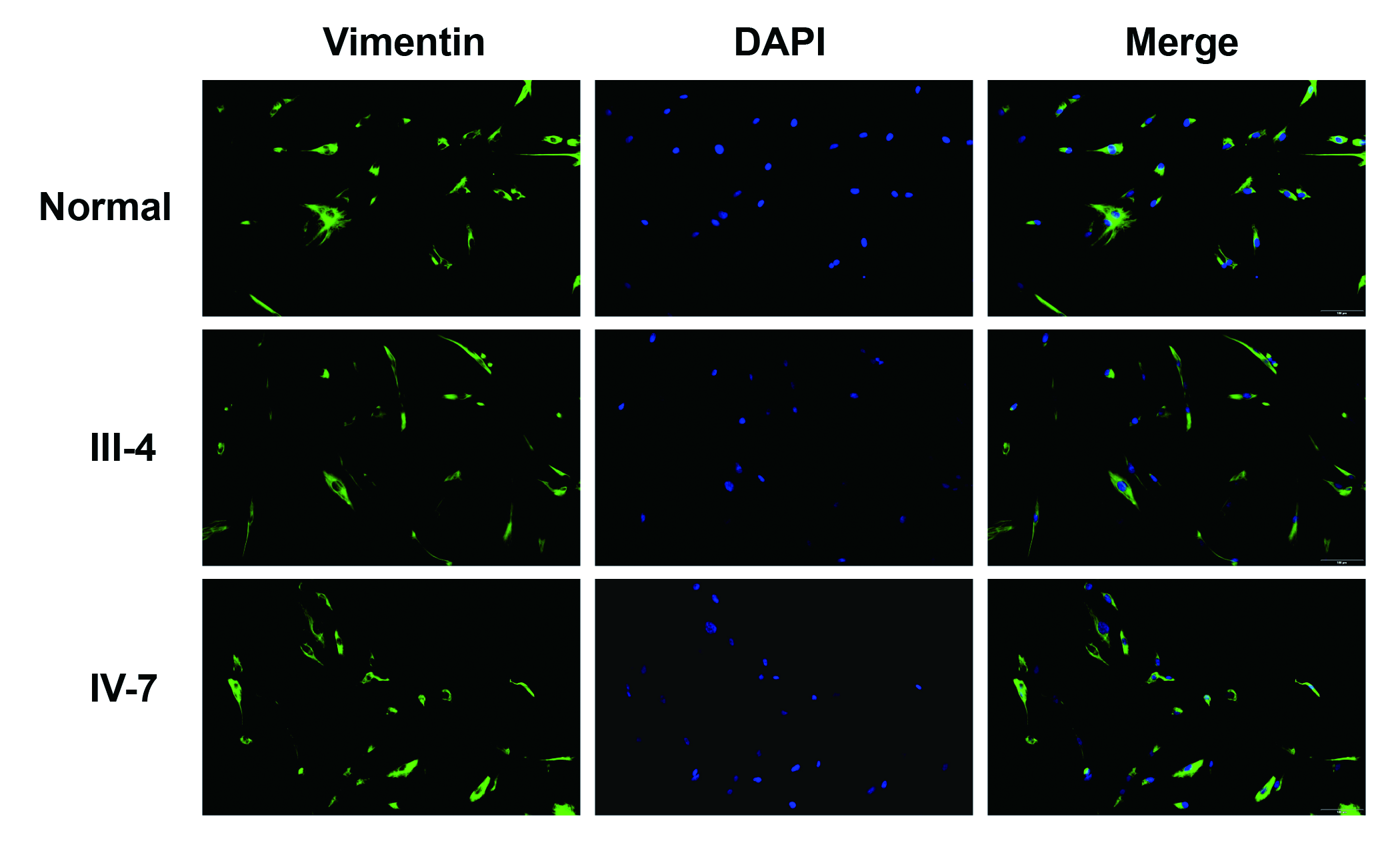

Supplement: Supplementary file 7 — Supplementary figure 6 [file 41420_2022_1160_MOESM7_ESM.tif]
