# Supplementary material for: A novel mutation located in the intermembrane space domain of AFG3L2 causes dominant optic atrophy through decreasing the stability of the encoded protein
Source: Cell Death Discov. 2022 Aug 15;8:361. doi: 10.1038/s41420-022-01160-9 (PMC9378676; doi:10.1038/s41420-022-01160-9)
Supplement: Supplementary file 8 — STR analysis of HEK293T [file 41420_2022_1160_MOESM8_ESM.docx]

1. **细胞遗传质量鉴定检测**
2. **Cell Line Authentication Service**
3. **STR基因型检测报告**
4. **送检单位：葵赛**
5. **检品名称：**细胞系
6. **委托单位：**上海翼和应用生物技术有限公司
7. **报告日期：2022-04-26**

**报 告 说 明**

1. 本报告只对送检的来样负责。
2. 检验报告上的检验结果和检验单位名称，未经同意不得用于广告、评优及商业宣传。
3. 对本报告有异议，请于收到报告之日起十五日内以书面方式提出，逾期不予受理。
4. 对纸质检验报告涂改、增删，或未加盖检验单位印章的复印件均无效。

**样 品 信 息**

**样品编号：**

| 客户样本编号 | 公司编号 |
| --- | --- |
| 293T-P9 | 20220424-01 |
| 293-P7 | 20220424-02 |

**样品数量：**2

**样品性状：**细胞系

**检测项目：**STR

**送检单位：葵赛**

**检测方法：**用Axygen的基因组抽提试剂盒提取DNA，采用21- STR扩增方案扩增，在ABI 3730XL型遗传分析仪上对STR位点和性别基因Amelogenin进行检测。

**检 测 结 果**

1. **检验基本情况**

| **公司编号** | **多等位基因** | **匹配细胞系** | **细胞库** | **EV值** | **匹配说明** |
| --- | --- | --- | --- | --- | --- |
| 20220424-01 | 无 | HEK293T | EXPASY | 0.94 | 基本匹配 |
| 20220424-02 | 无 | HEK293T | EXPASY | 0.94 | 基本匹配 |

样本基因型检验结果

- 多等位基因指三等位及以上基因现象。
- 本次检测各细胞分型结果良好。

1. **各样本描述**

- 20220424-01：该株细胞DNA分型在细胞系检索中找到**基本匹配**的细胞系，EXPASY数据库显示细胞名为**HEK293T**，细胞号对应**CVCL_0063**。本次检测在该细胞系中**没有发现多等位基因**。


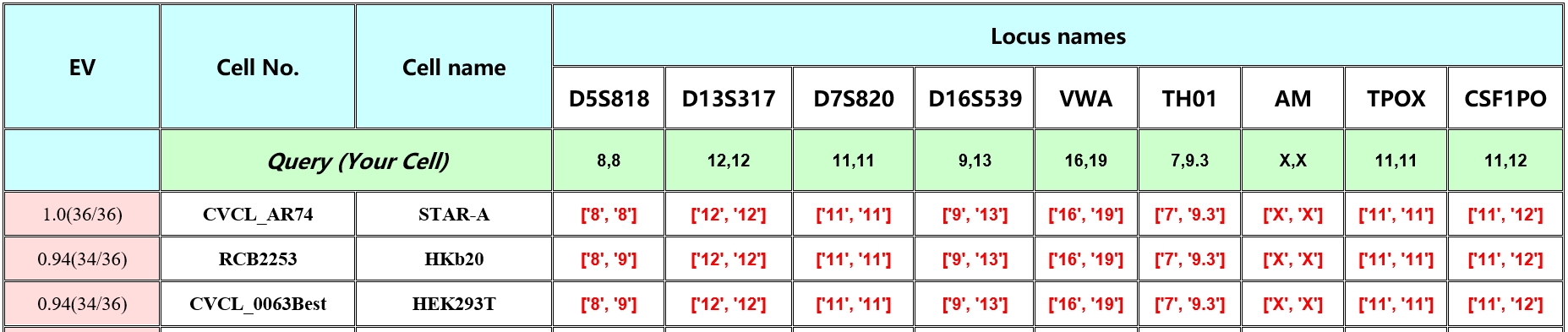


- 20220424-02：该株细胞DNA分型在细胞系检索中找到**基本匹配**的细胞系，EXPASY数据库显示细胞名为**HEK293T**，细胞号对应**CVCL_0063**。本次检测在该细胞系中**没有发现多等位基因**。


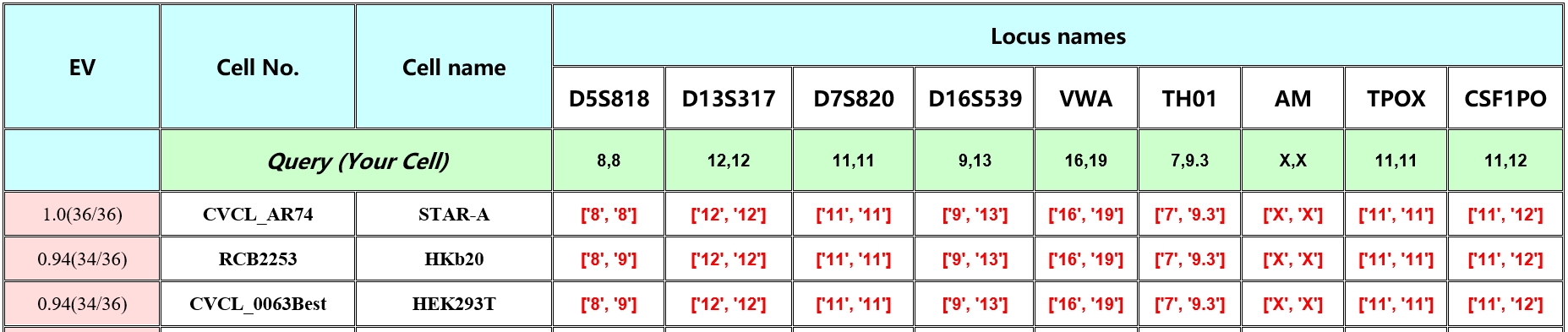


**备注：**待测细胞系与收录于ATCC, DSMZ, JCRB 和 RIKEN数据库的细胞系STR数据进行比对，未收录于以上细胞库的细胞系将无法匹配。

1. **样本分型结果**

| **细胞的STR位点和Amelogenin位点的基因分型结果** | | | | | | |
| --- | --- | --- | --- | --- | --- | --- |
| Loci | 送检细胞STR信息 | | | 细胞库细胞STR信息 | | |
|  | 送检细胞名：293T-P9 | | | 细胞库细胞名： HEK293T | | |
|  | Allele1 | Allele2 | Allele3 | Allele1 | Allele2 | Allele3 |
| D5S818 | 8 | 8 |  | 8 | 9 |  |
| D13S317 | 12 | 12 |  | 12 | 12 |  |
| D7S820 | 11 | 11 |  | 11 | 11 |  |
| D16S539 | 9 | 13 |  | 9 | 13 |  |
| VWA | 16 | 19 |  | 16 | 19 |  |
| TH01 | 7 | 9.3 |  | 7 | 9.3 |  |
| AMEL | X | X |  | X | X |  |
| TPOX | 11 | 11 |  | 11 | 11 |  |
| CSF1PO | 11 | 12 |  | 11 | 12 |  |
| D12S391 | 19 | 21 |  |  |  |  |
| FGA | 23 | 23 |  |  |  |  |
| D2S1338 | 19 | 19 |  |  |  |  |
| D21S11 | 28 | 28 |  |  |  |  |
| D18S51 | 17 | 18 |  |  |  |  |
| D8S1179 | 12 | 12 |  |  |  |  |
| D3S1358 | 17 | 17 |  |  |  |  |
| D6S1043 | 11 | 11 |  |  |  |  |
| PENTAE | 7 | 15 |  |  |  |  |
| D19S433 | 18.2 | 18.2 |  |  |  |  |
| PENTAD | 9 | 10 |  |  |  |  |
| D1S1656 | 15 | 17.3 |  |  |  |  |

| **细胞的STR位点和Amelogenin位点的基因分型结果** | | | | | | |
| --- | --- | --- | --- | --- | --- | --- |
| Loci | 送检细胞STR信息 | | | 细胞库细胞STR信息 | | |
|  | 送检细胞名：293-P7 | | | 细胞库细胞名： HEK293T | | |
|  | Allele1 | Allele2 | Allele3 | Allele1 | Allele2 | Allele3 |
| D5S818 | 8 | 8 |  | 8 | 9 |  |
| D13S317 | 12 | 12 |  | 12 | 12 |  |
| D7S820 | 11 | 11 |  | 11 | 11 |  |
| D16S539 | 9 | 13 |  | 9 | 13 |  |
| VWA | 16 | 19 |  | 16 | 19 |  |
| TH01 | 7 | 9.3 |  | 7 | 9.3 |  |
| AMEL | X | X |  | X | X |  |
| TPOX | 11 | 11 |  | 11 | 11 |  |
| CSF1PO | 11 | 12 |  | 11 | 12 |  |
| D12S391 | 19 | 21 |  |  |  |  |
| FGA | 23 | 23 |  |  |  |  |
| D2S1338 | 19 | 19 |  |  |  |  |
| D21S11 | 28 | 28 |  |  |  |  |
| D18S51 | 17 | 18 |  |  |  |  |
| D8S1179 | 12 | 12 |  |  |  |  |
| D3S1358 | 17 | 17 |  |  |  |  |
| D6S1043 | 11 | 11 |  |  |  |  |
| PENTAE | 7 | 15 |  |  |  |  |
| D19S433 | 18.2 | 18.2 |  |  |  |  |
| PENTAD | 9 | 10 |  |  |  |  |
| D1S1656 | 15 | 17.3 |  |  |  |  |

**其 他 说 明**

1. **分型方案及位点分布**

|  | **方案1** | **方案2** | **方案3** | **方案4** |
| --- | --- | --- | --- | --- |
| 1 | D3S1358 | D8S1179 | D19S433 | AMEL |
| 2 | VWA | D21S11 | TH01 | D1S1656 |
| 3 | D7S820 | D16S539 | D13S317 | D5S818 |
| 4 | CSF1PO | D2S1338 | TPOX | D12S391 |
| 5 | PENTAE | PENTAD | D18S51 | FGA |
| 6 |  |  | D6S1043 |  |

实验方案及位点

1. **STR数据库比对**

本公司采用DSMZ tools进行细胞系比对，其中包含来自于ATCC, DSMZ, JCRB 和 RIKEN数据库的2455个细胞系STR数据。如果待检测细胞未收录于以上细胞库或这是自行建立的新细胞系将无法进行比对，用户需根据细胞分型结果自行与其他数据库进行比对。

1. **文献引用参考**

1．Authentication testing of HEK 293T and HeLa cell lines have been performed by Shanghai Biowing Applied Biotechnology Co.,Ltd via STR profiling. STR profiles match the standards recommended for HEK 293T and HeLa cell lines authentication

2．AGS, NCI-N87, HGC-27 and HEK293 were STR-authenticated on Dec. 8, 2015 by Shanghai Biowing Applied Biotechnology Co. LTD, Shanghai, China

**主要实验人员：**张佳男

**复核人:** 张晨茜

**负责人：**巢凯悦

**签发日期：2022-04-26**
