# Supplementary material for: A novel mutation located in the intermembrane space domain of AFG3L2 causes dominant optic atrophy through decreasing the stability of the encoded protein
Source: Cell Death Discov. 2022 Aug 15;8:361. doi: 10.1038/s41420-022-01160-9 (PMC9378676; doi:10.1038/s41420-022-01160-9)
Supplement: Supplementary file 9 — Original Data File [file 41420_2022_1160_MOESM9_ESM.pptx]

## Slide 1
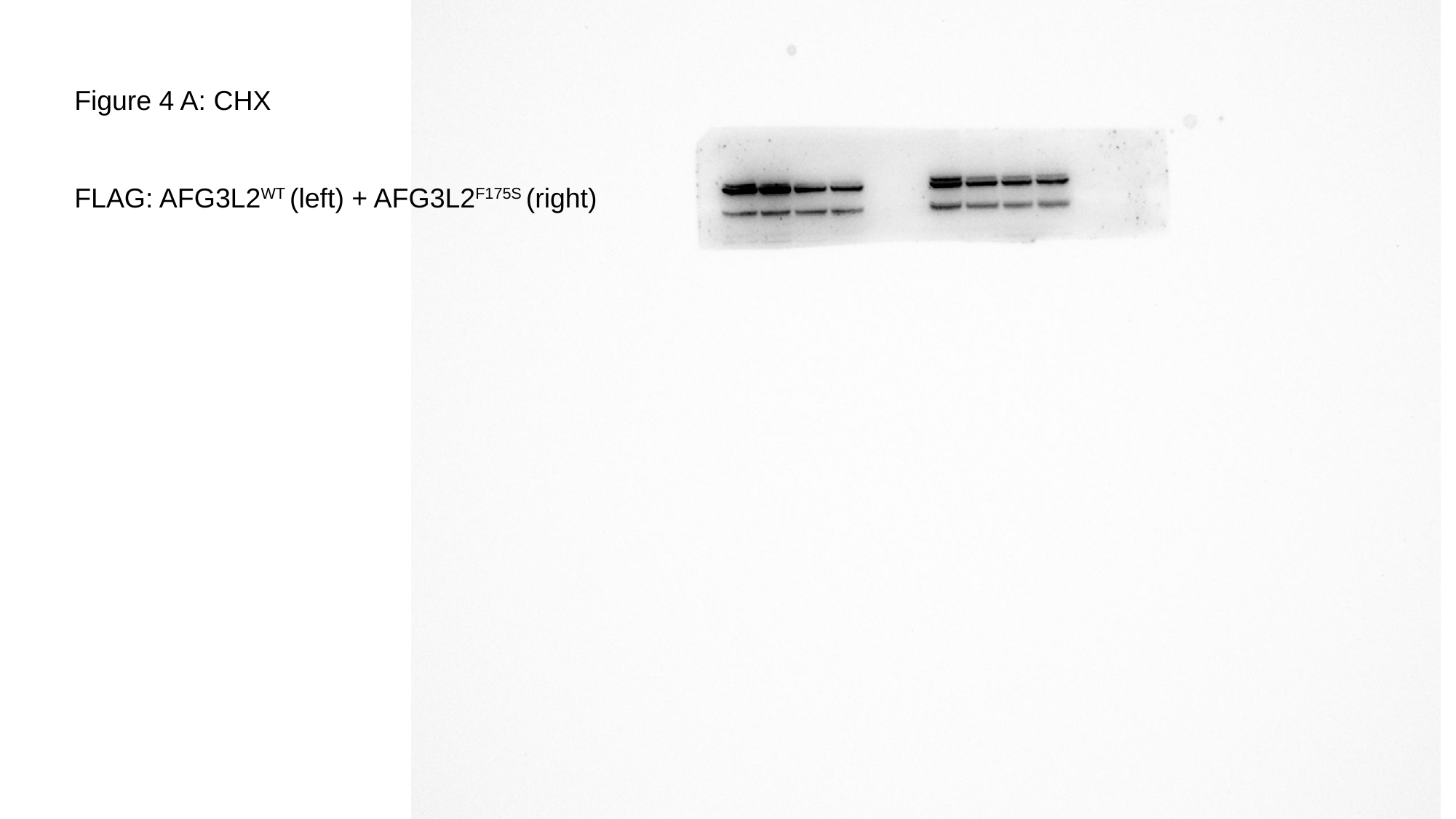

Figure 4 A: CHX
FLAG: AFG3L2WT (left) + AFG3L2F175S (right)

## Slide 2
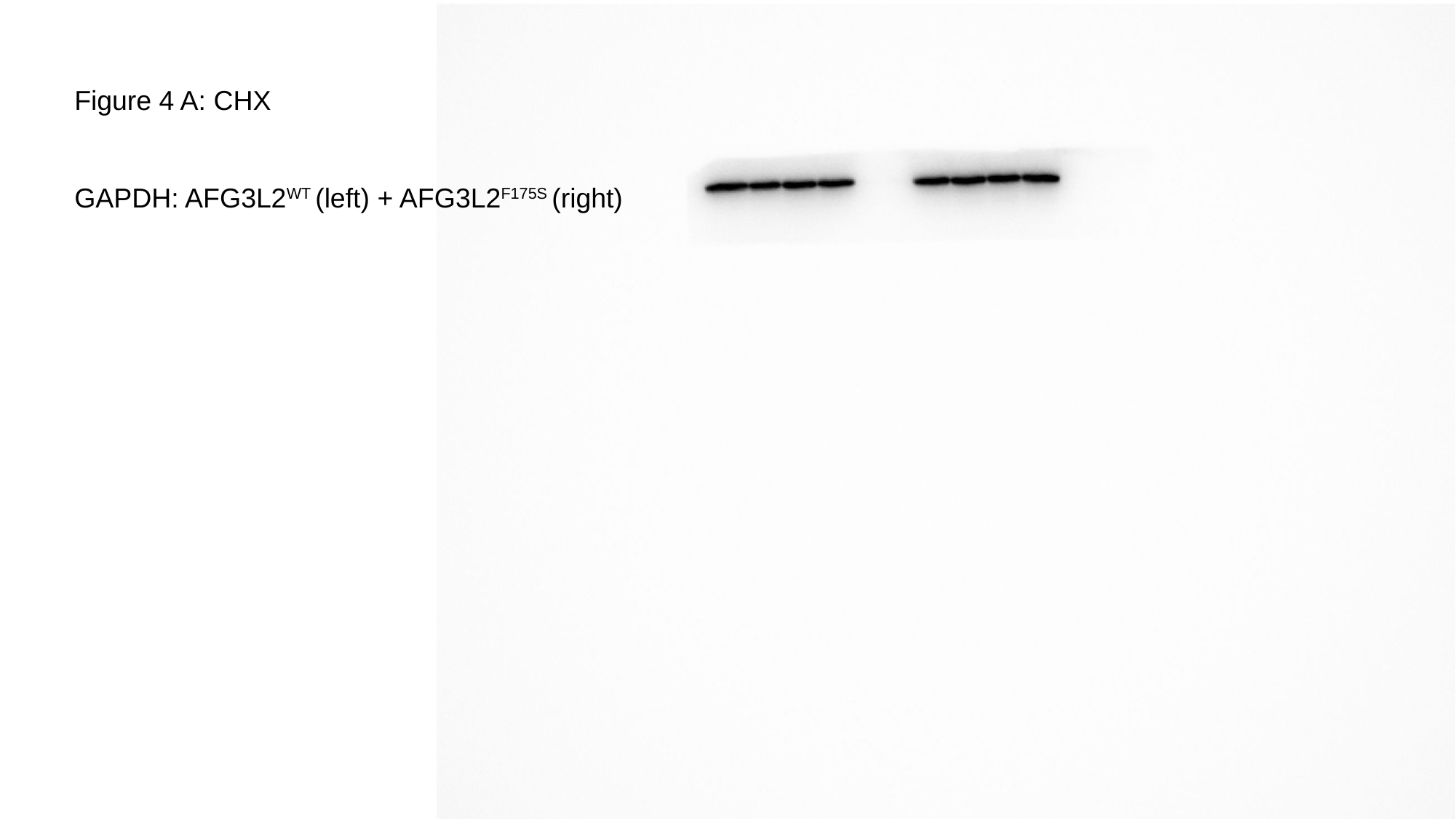

Figure 4 A: CHX
GAPDH: AFG3L2WT (left) + AFG3L2F175S (right)

## Slide 3
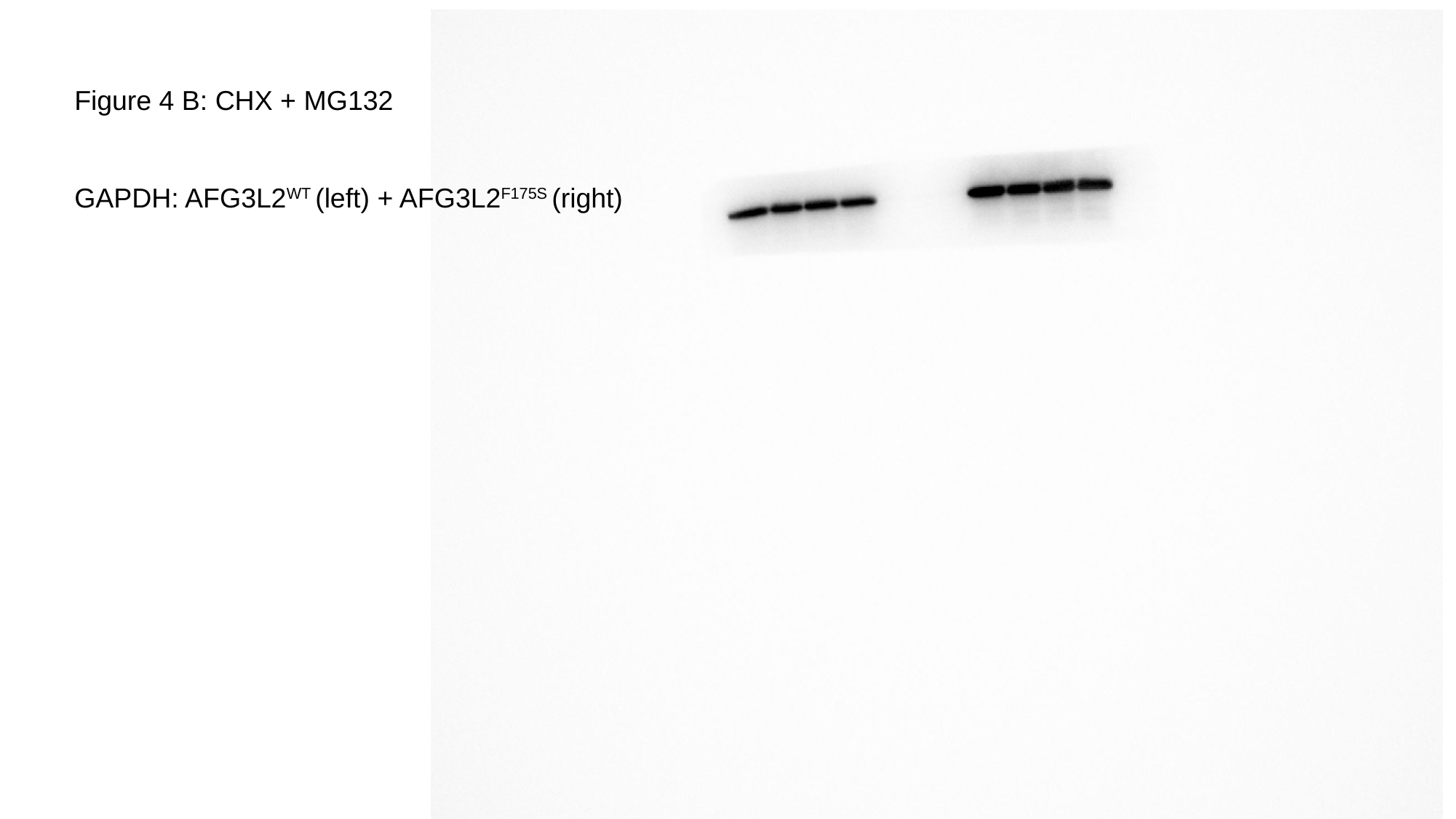

Figure 4 B: CHX + MG132
GAPDH: AFG3L2WT (left) + AFG3L2F175S (right)

## Slide 4
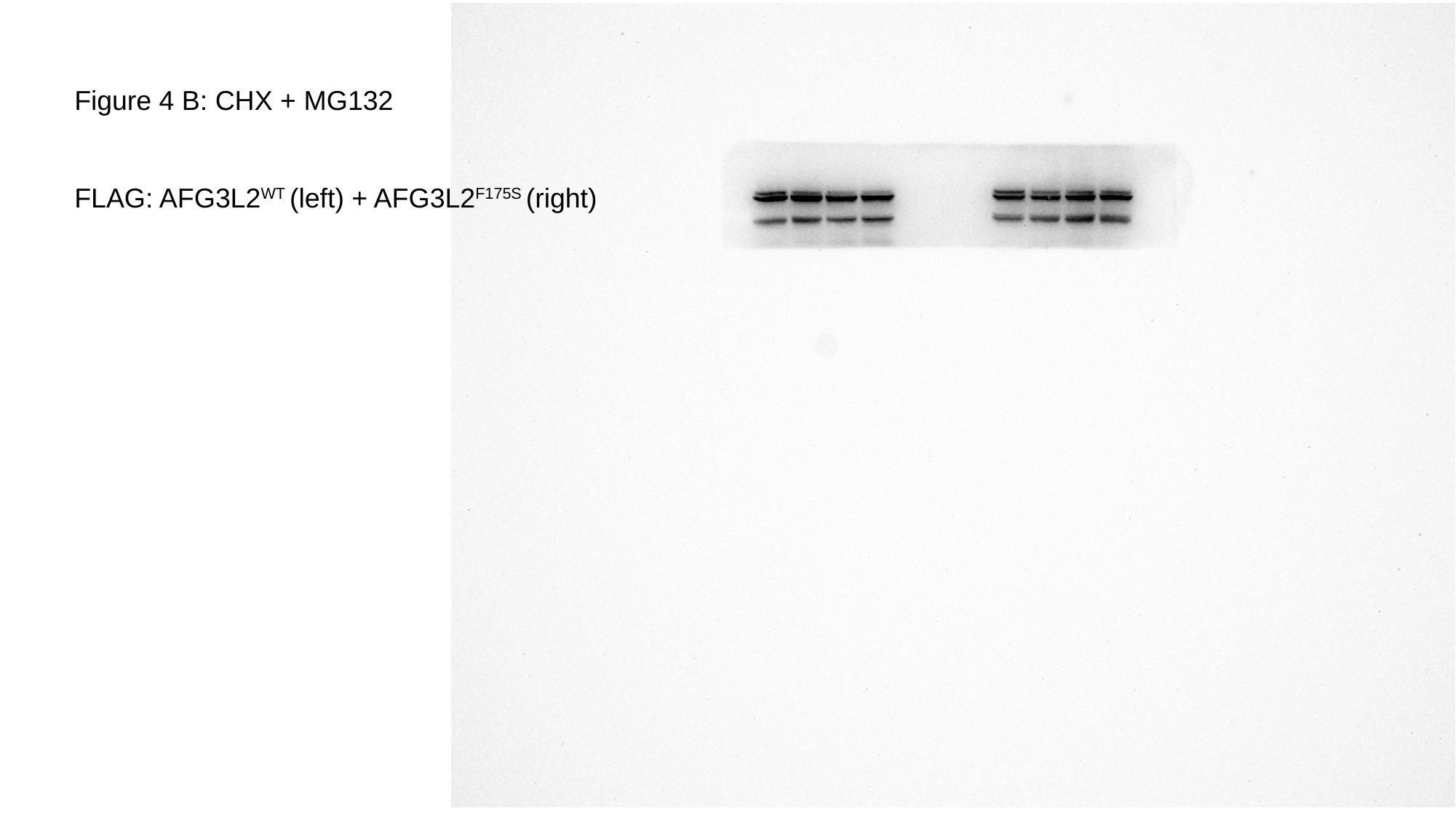

Figure 4 B: CHX + MG132
FLAG: AFG3L2WT (left) + AFG3L2F175S (right)

## Slide 5
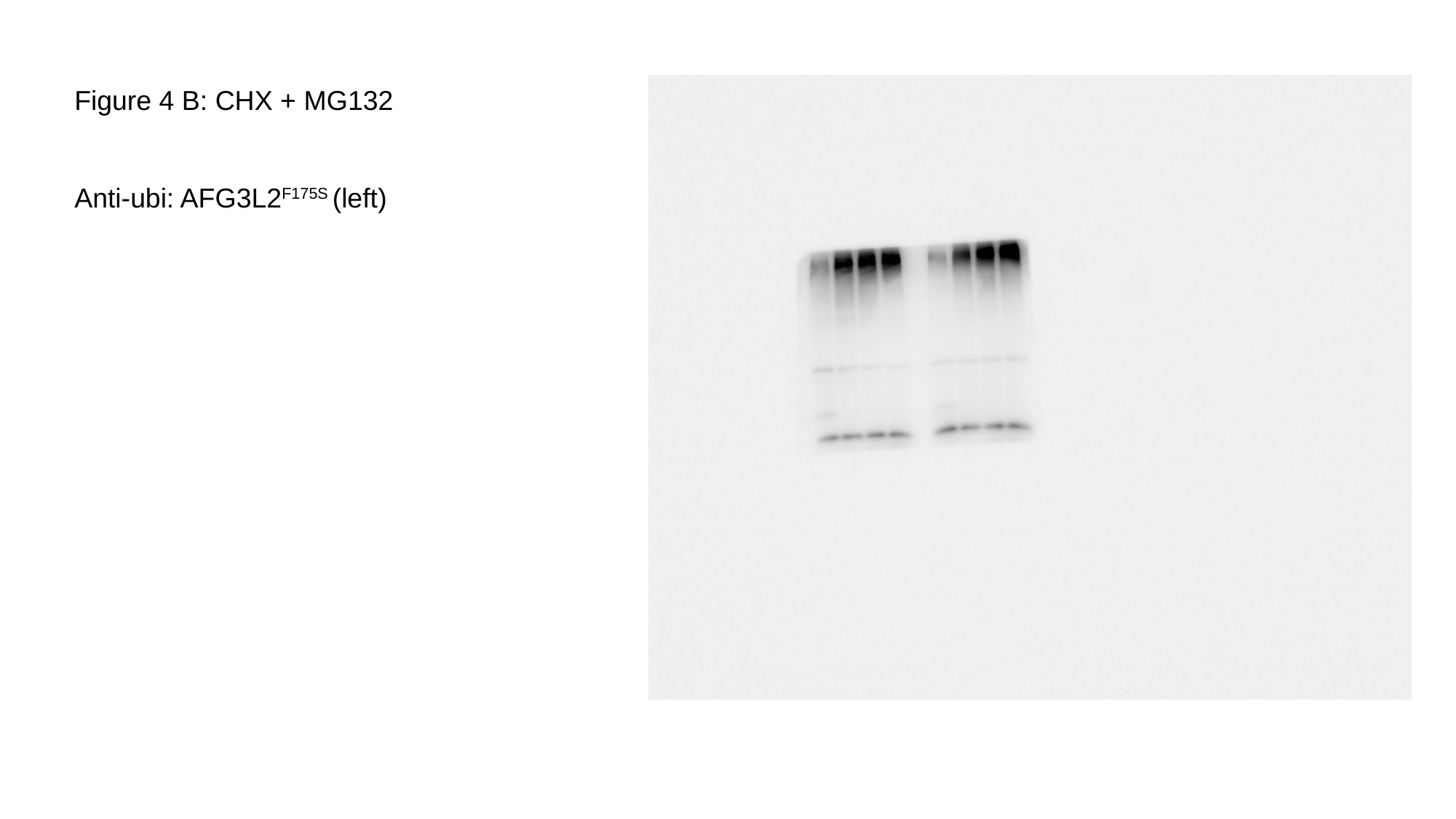

Figure 4 B: CHX + MG132
Anti-ubi: AFG3L2F175S (left)

## Slide 6
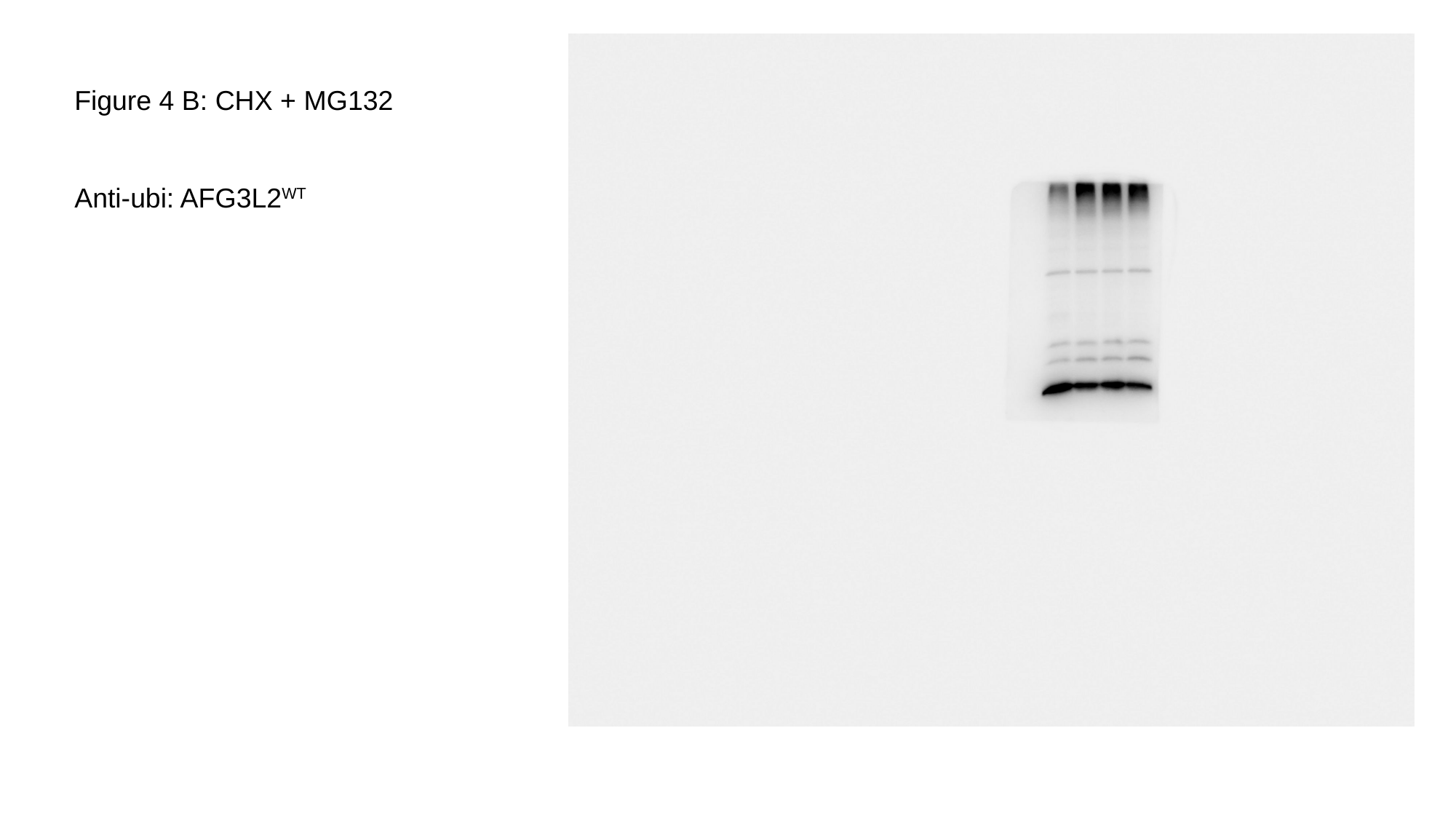

Figure 4 B: CHX + MG132
Anti-ubi: AFG3L2WT

## Slide 7
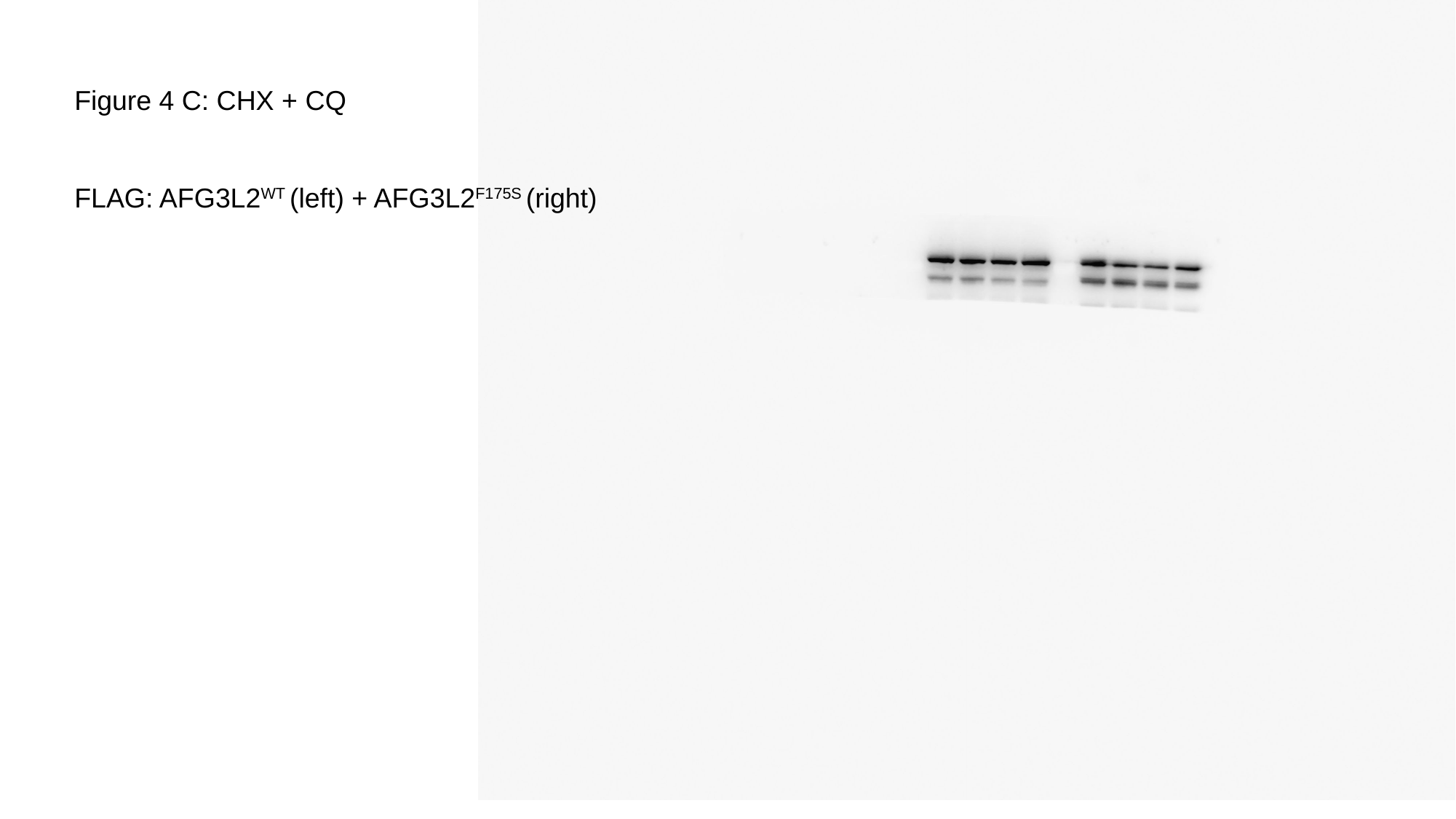

Figure 4 C: CHX + CQ
FLAG: AFG3L2WT (left) + AFG3L2F175S (right)

## Slide 8
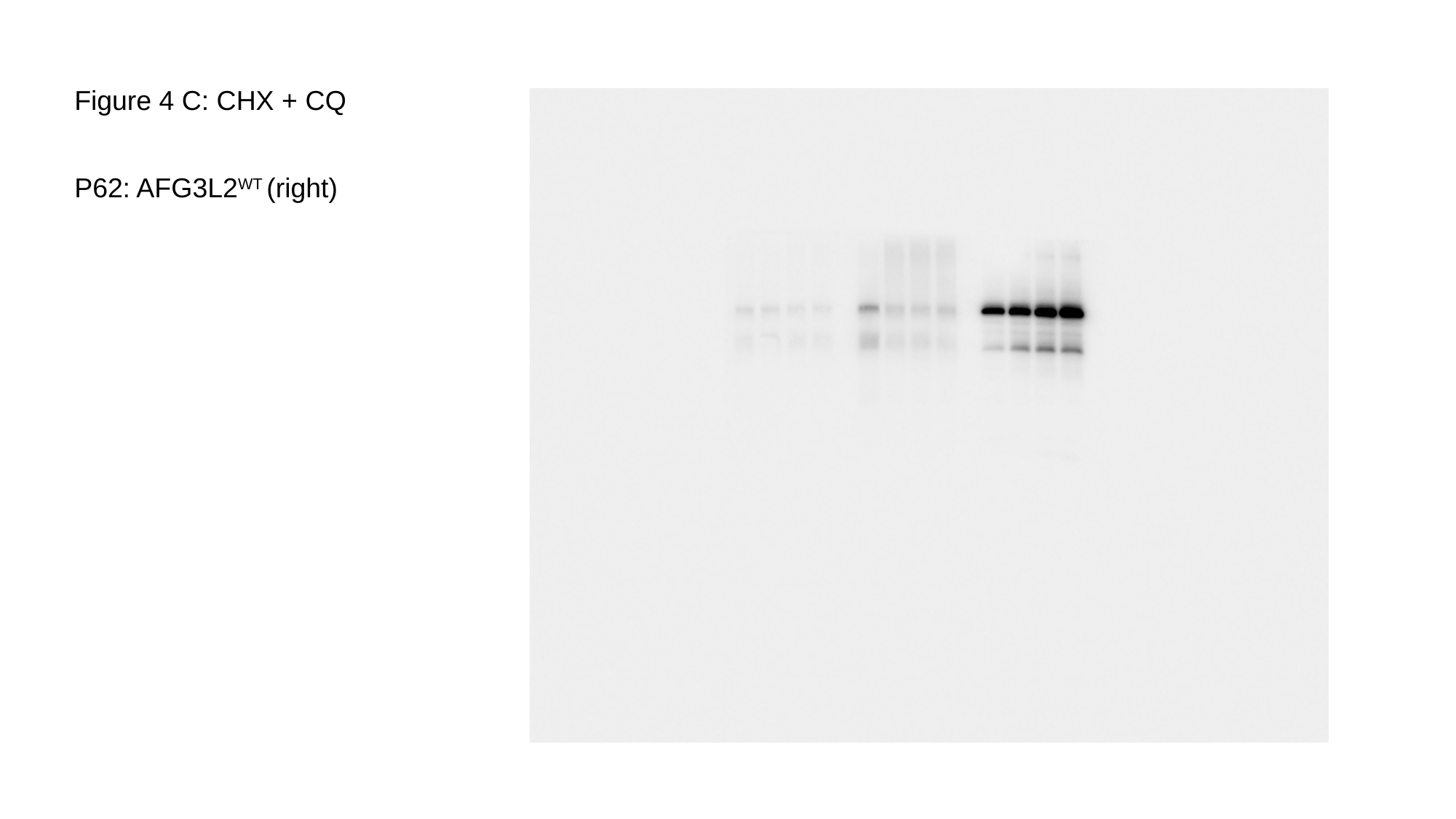

Figure 4 C: CHX + CQ
P62: AFG3L2WT (right)

## Slide 9
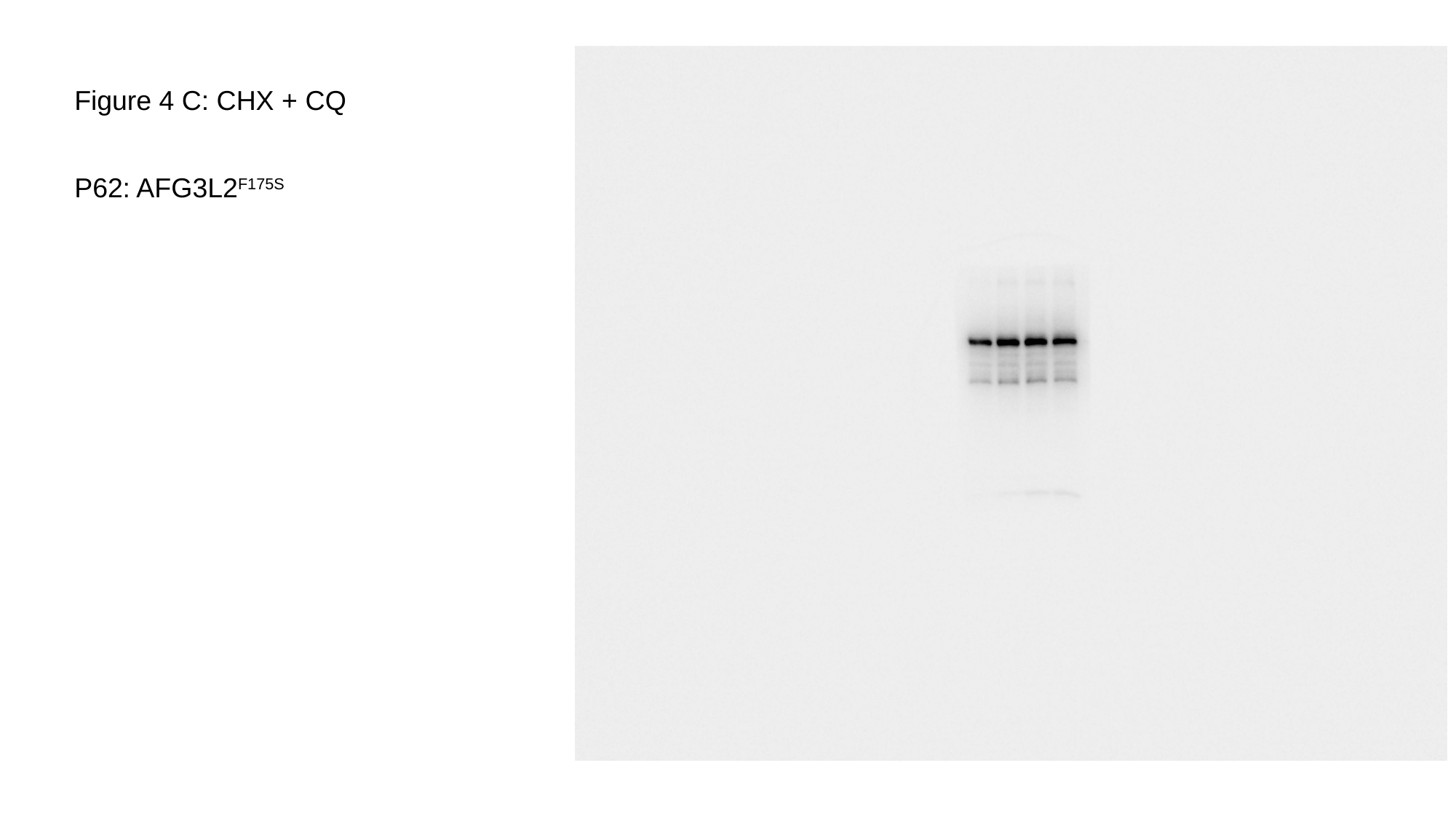

Figure 4 C: CHX + CQ
P62: AFG3L2F175S

## Slide 10
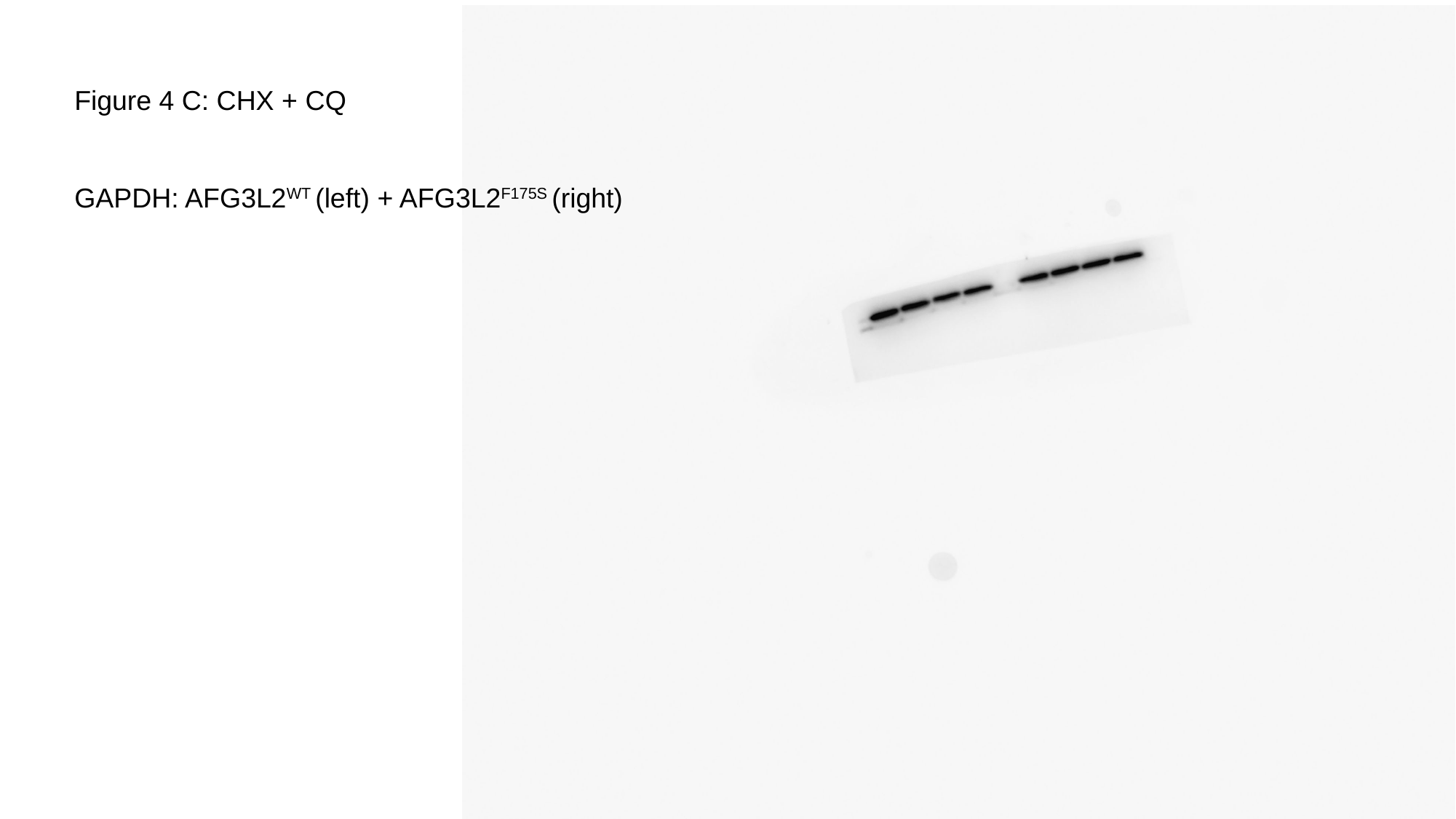

Figure 4 C: CHX + CQ
GAPDH: AFG3L2WT (left) + AFG3L2F175S (right)

## Slide 11
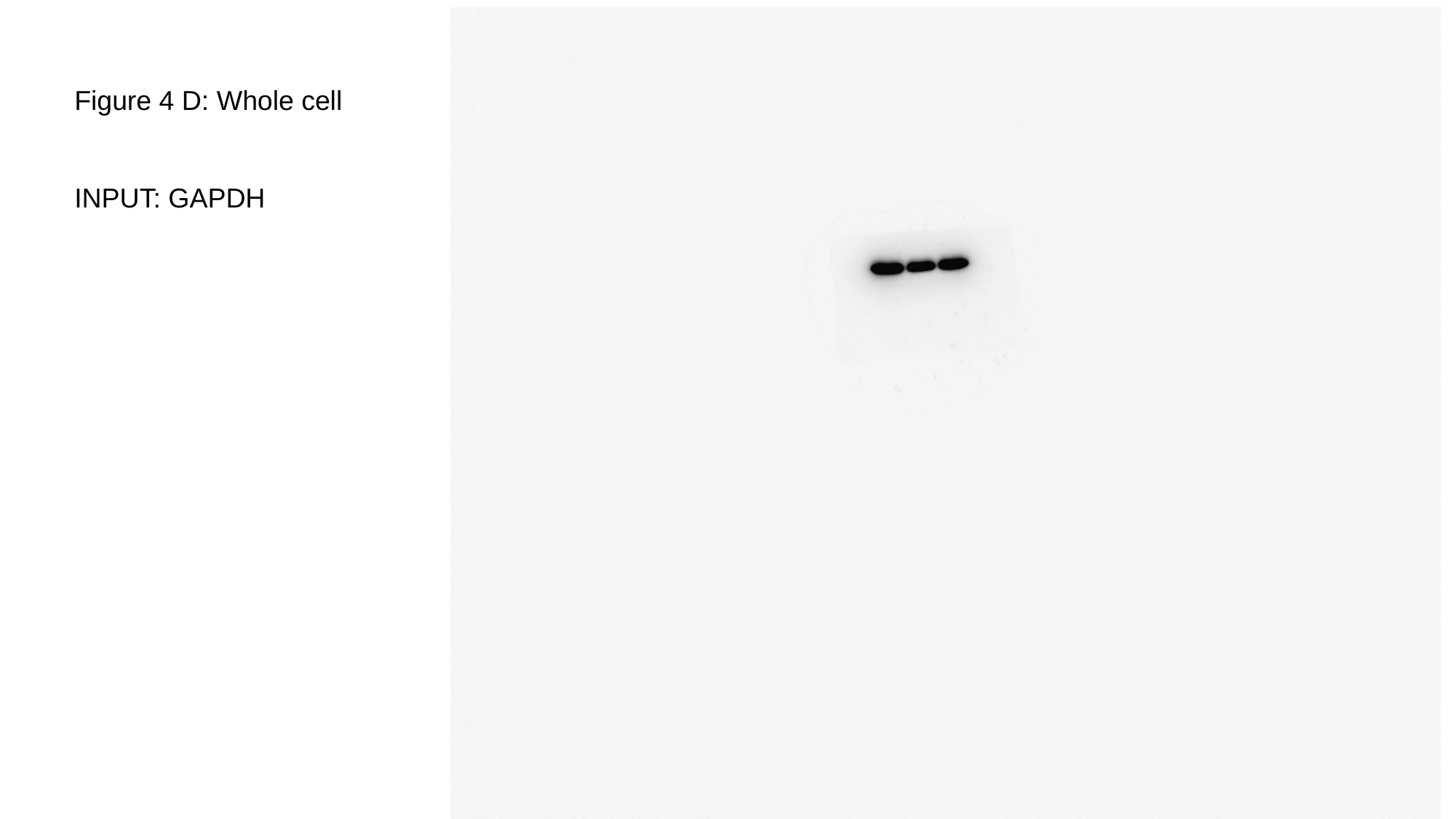

Figure 4 D: Whole cell
INPUT: GAPDH

## Slide 12
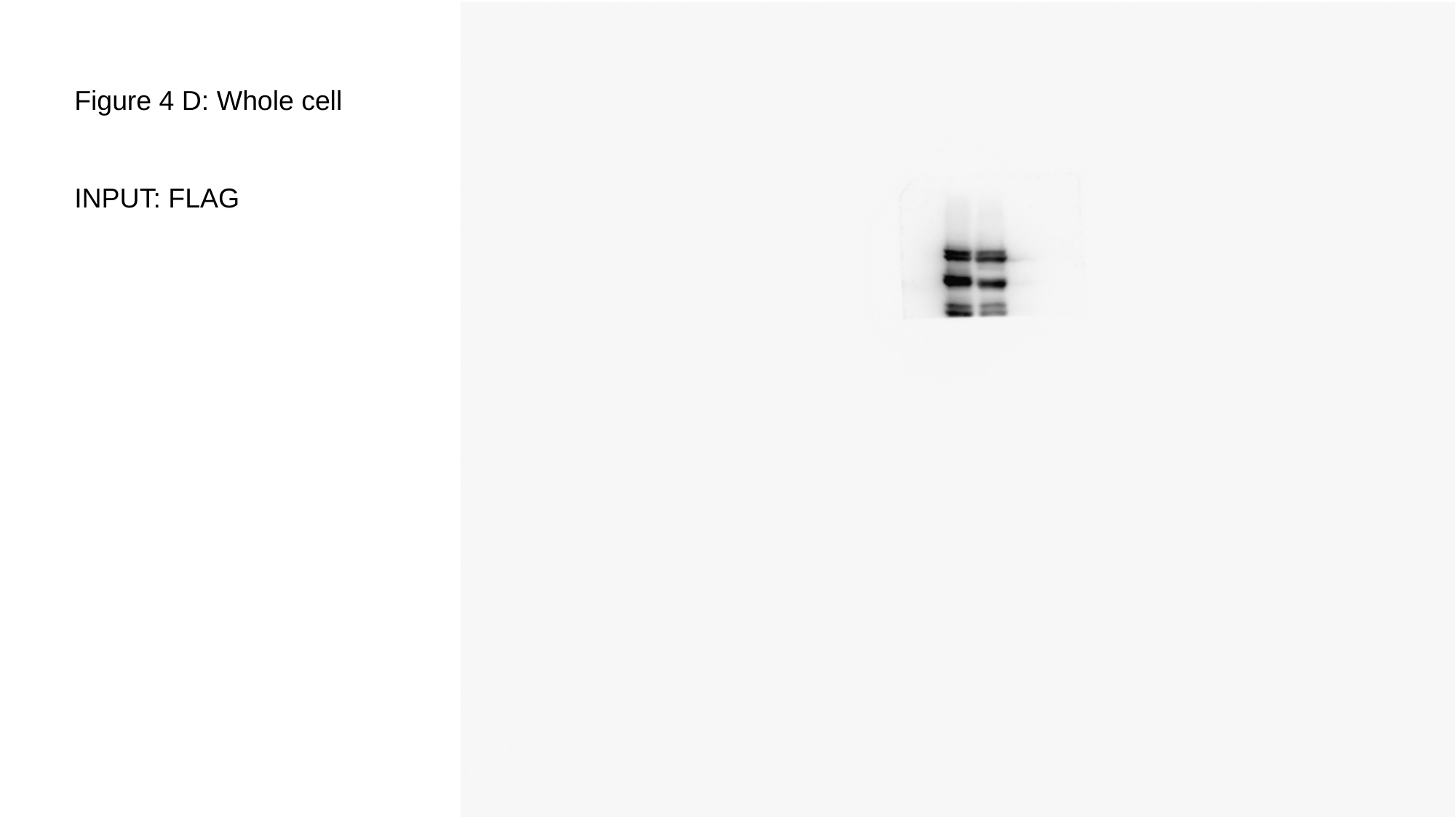

Figure 4 D: Whole cell
INPUT: FLAG

## Slide 13
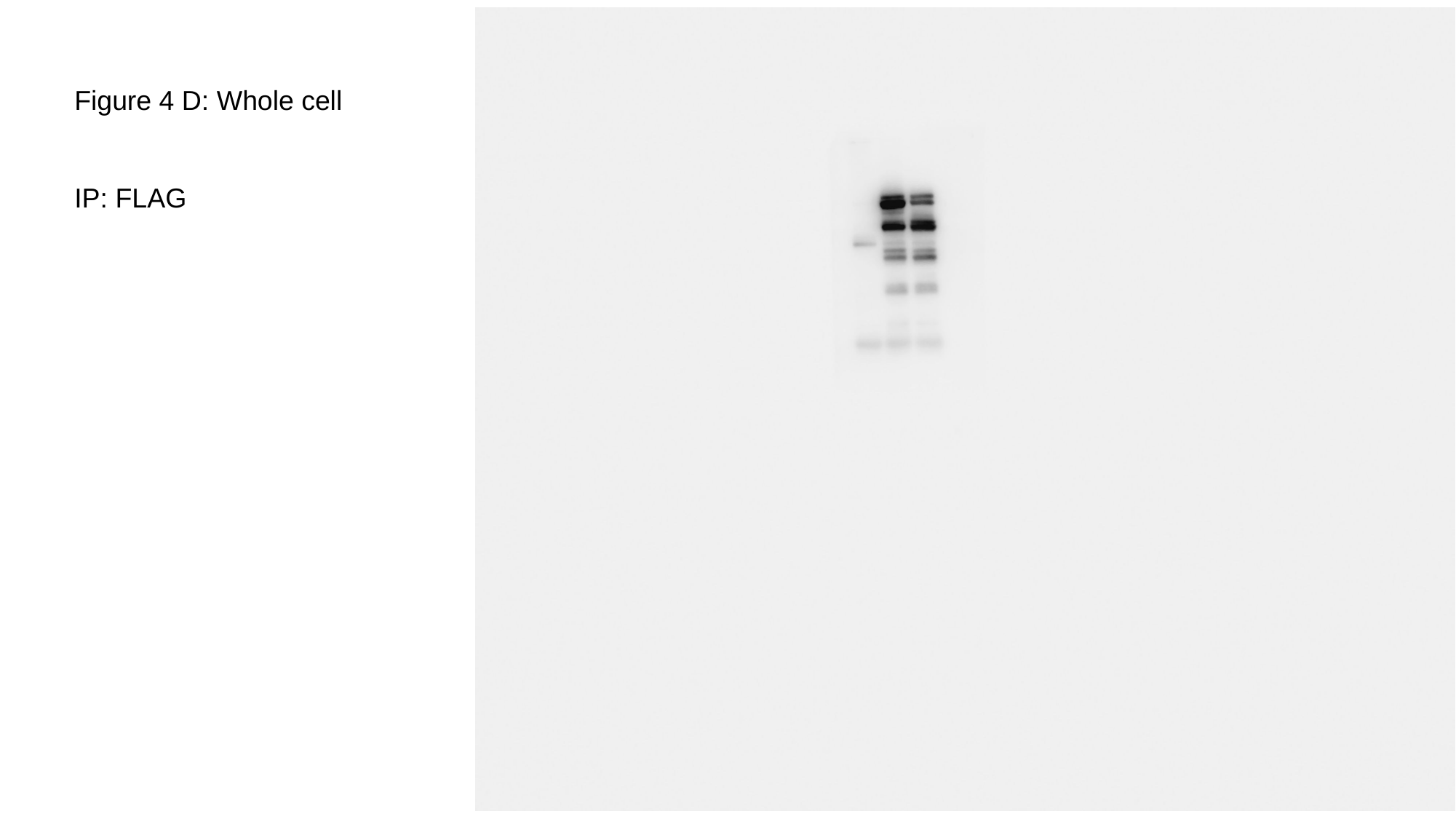

Figure 4 D: Whole cell
IP: FLAG

## Slide 14
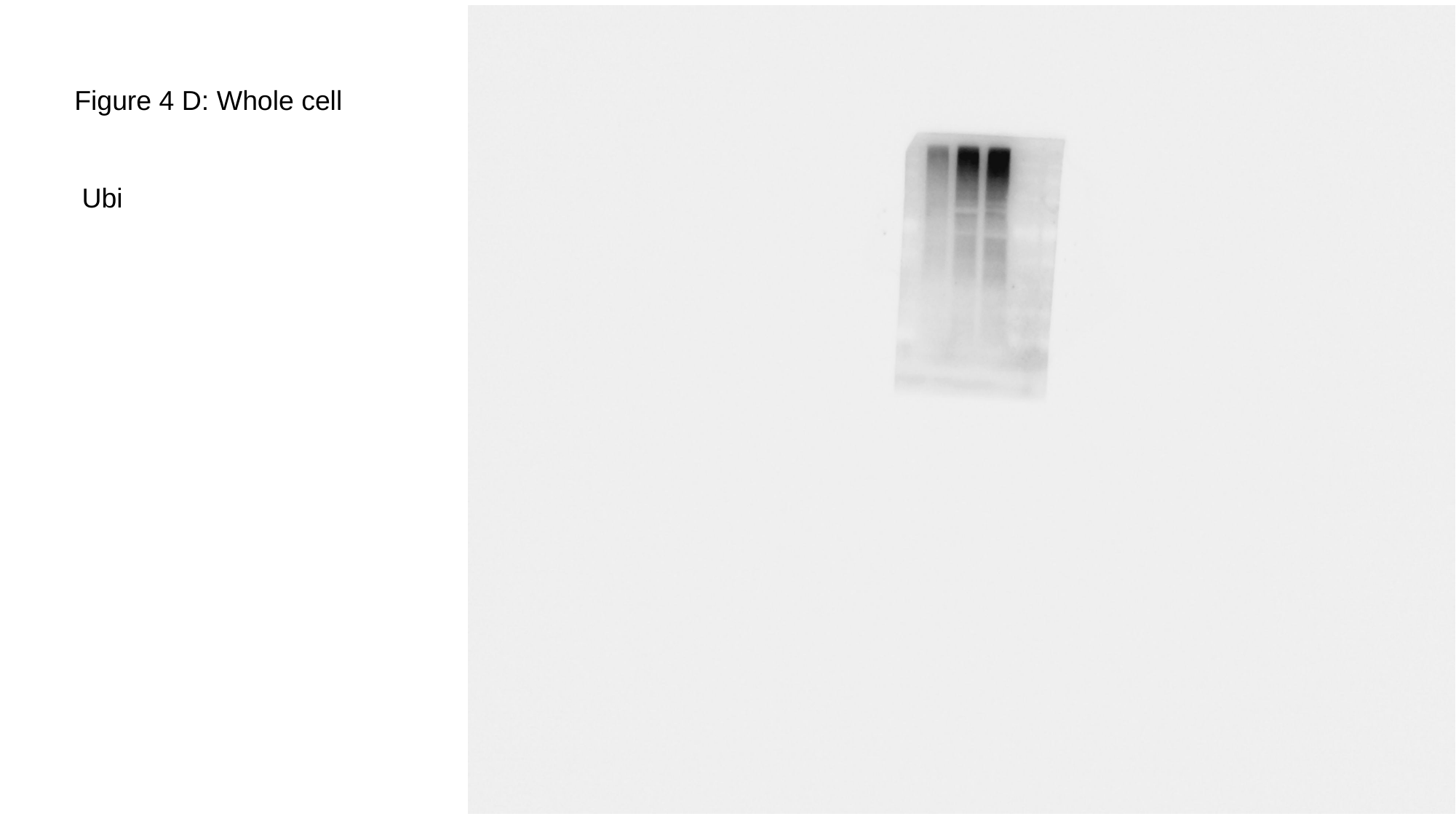

Figure 4 D: Whole cell
 Ubi

## Slide 15
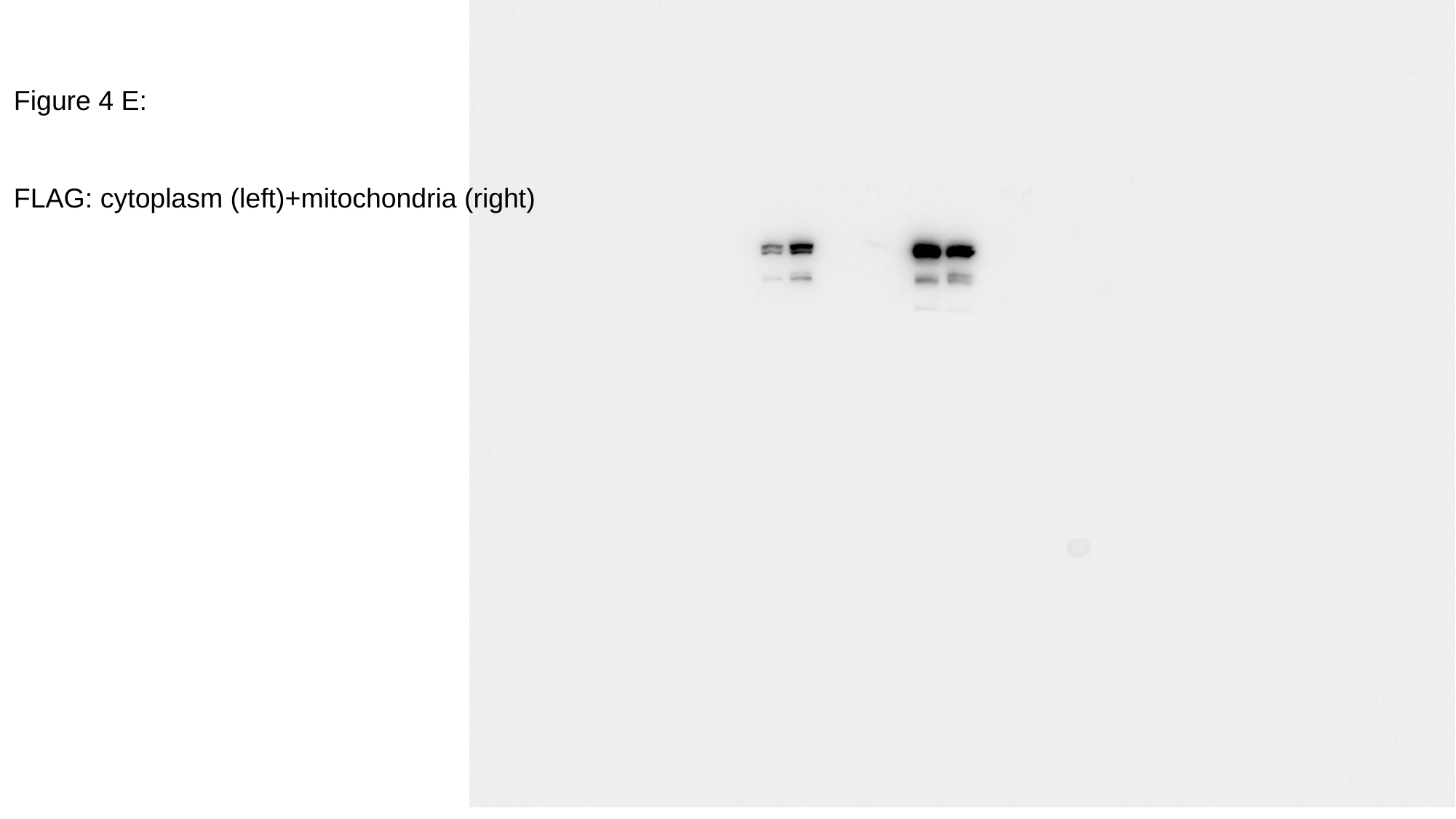

Figure 4 E:
FLAG: cytoplasm (left)+mitochondria (right)

## Slide 16
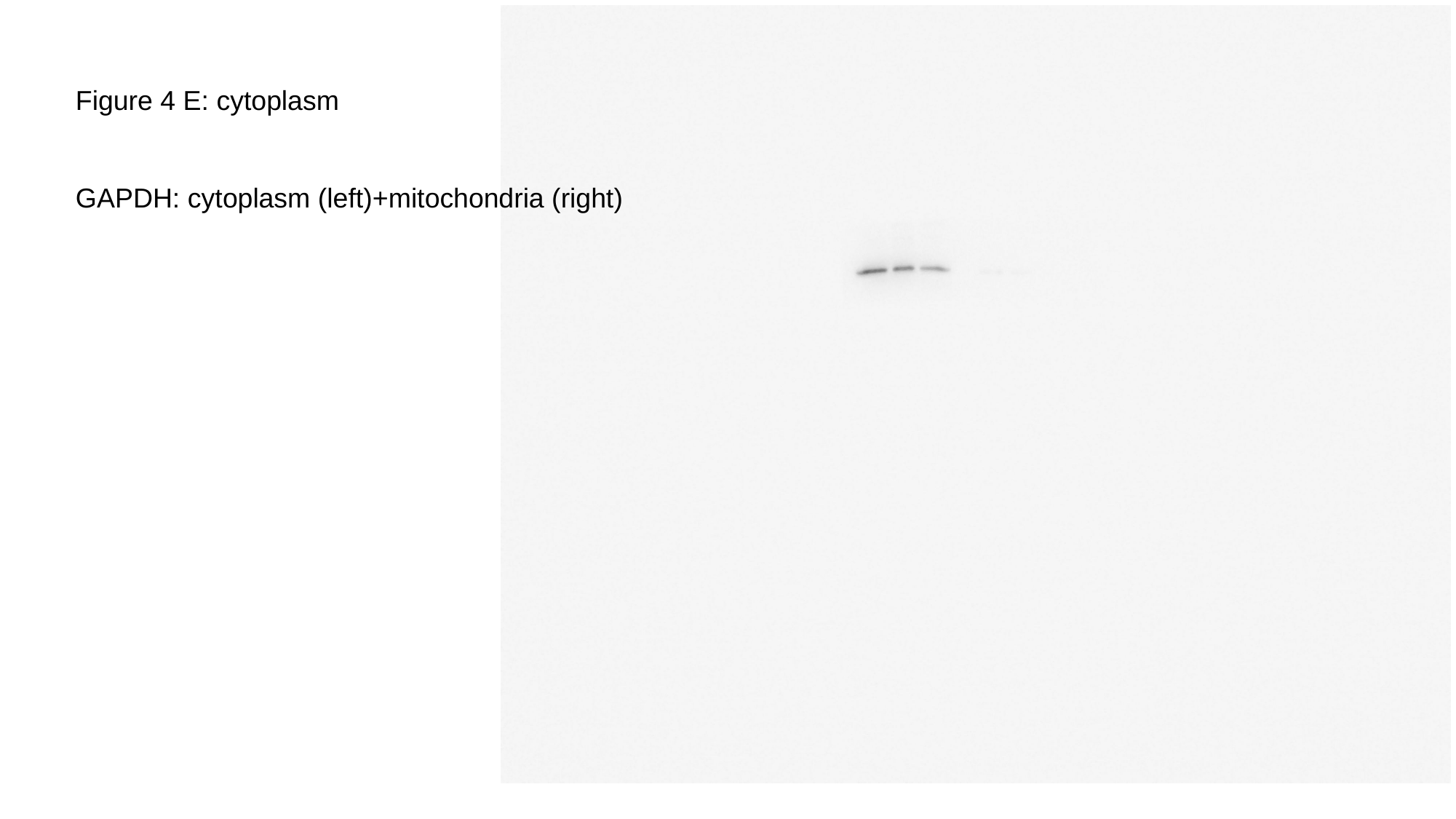

Figure 4 E: cytoplasm
GAPDH: cytoplasm (left)+mitochondria (right)

## Slide 17
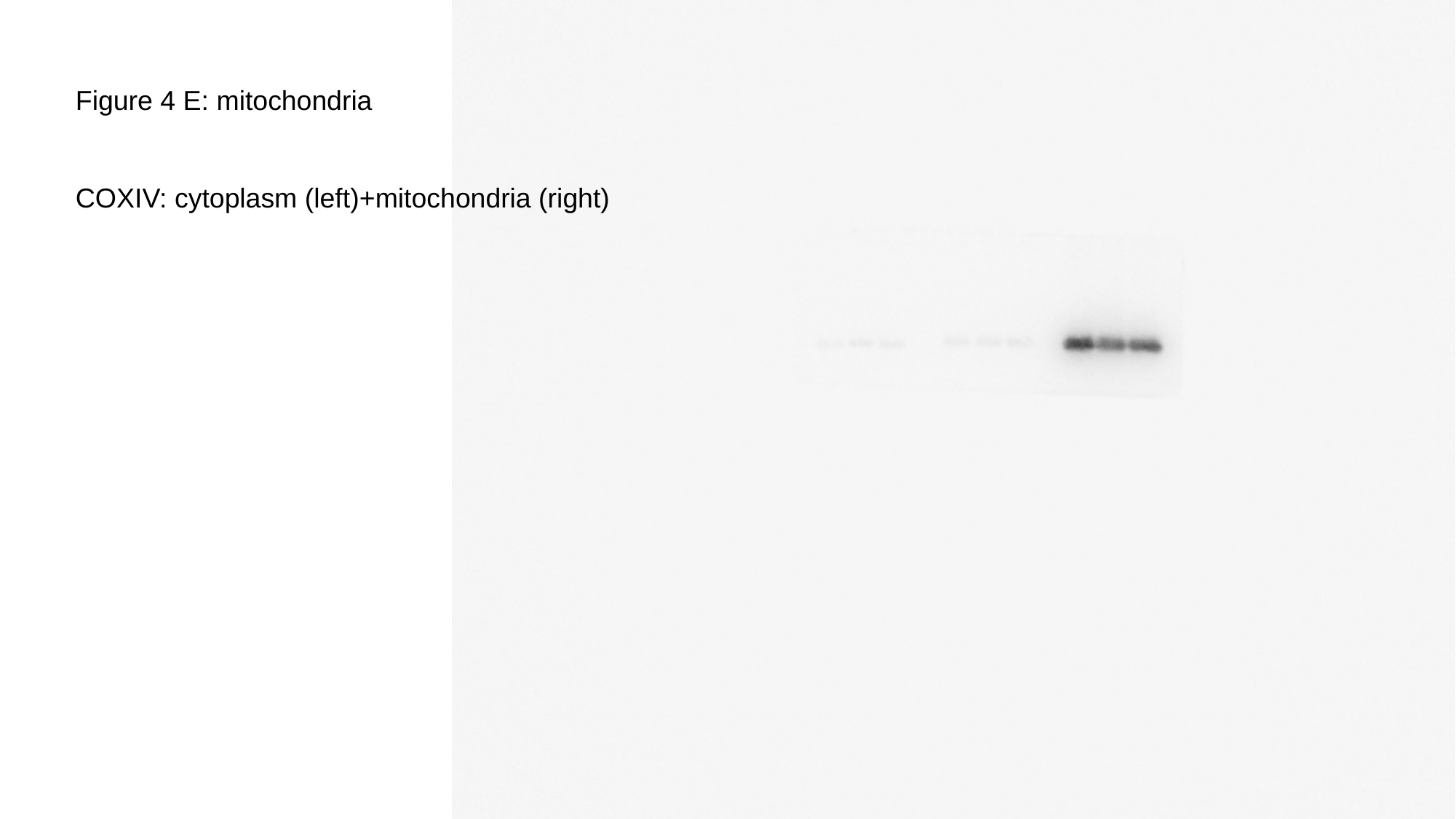

Figure 4 E: mitochondria
COXIV: cytoplasm (left)+mitochondria (right)

## Slide 18
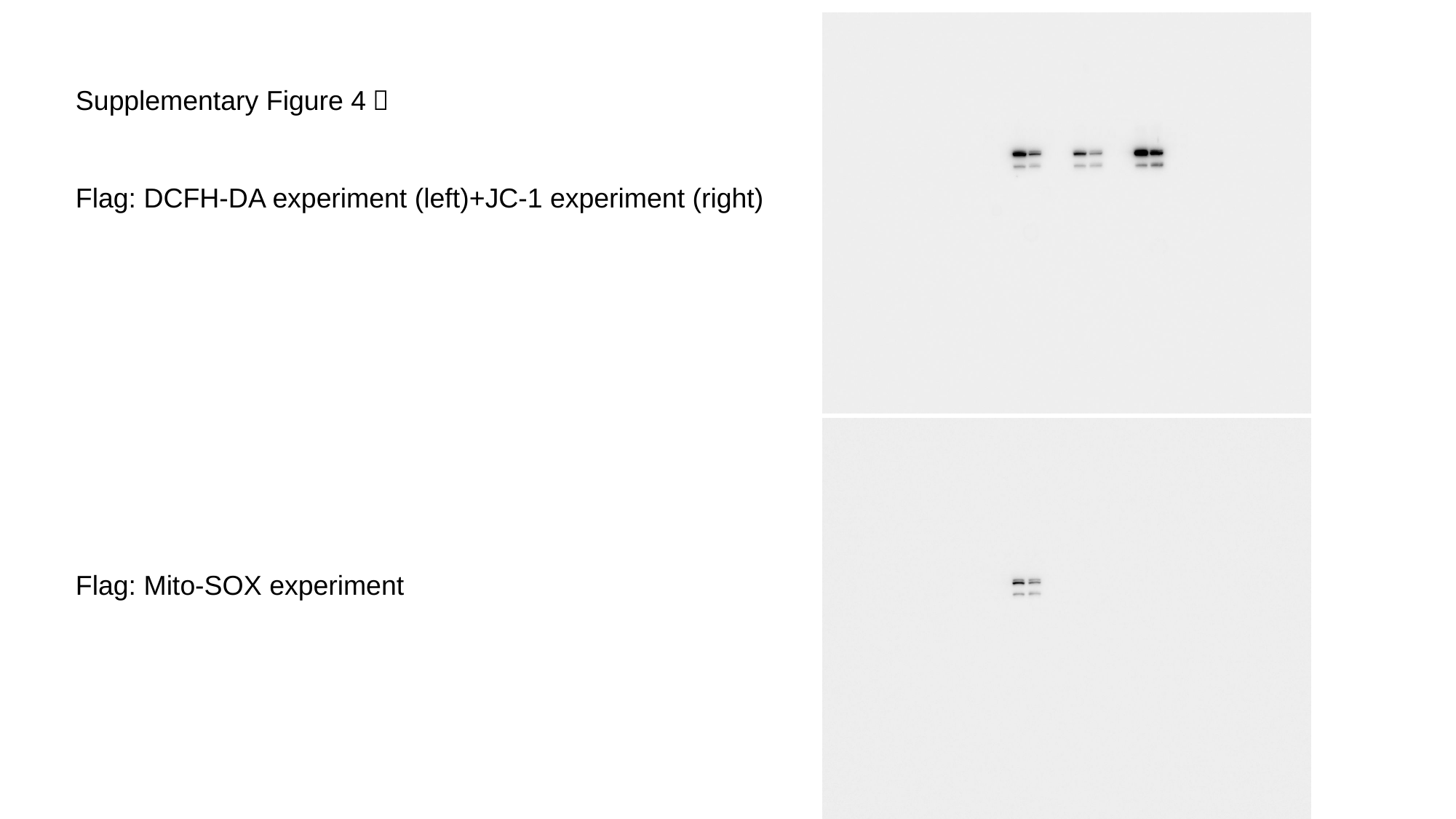

Supplementary Figure 4：
Flag: DCFH-DA experiment (left)+JC-1 experiment (right)
Flag: Mito-SOX experiment

## Slide 19
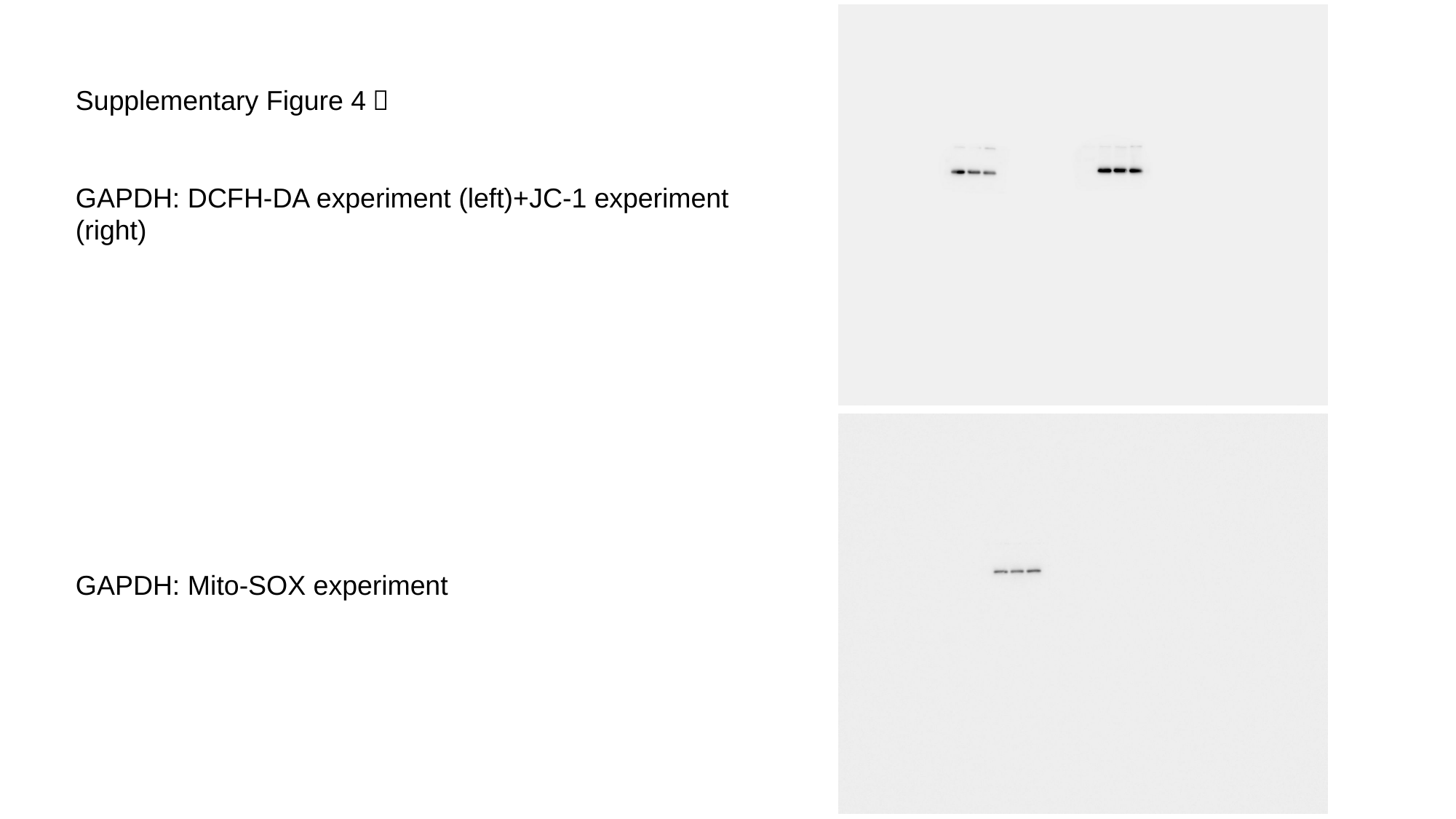

Supplementary Figure 4：
GAPDH: DCFH-DA experiment (left)+JC-1 experiment (right)
GAPDH: Mito-SOX experiment
